# Parameter redundancy in Jolly‐Seber tag loss models

**DOI:** 10.1002/ece3.7035

**Published:** 2021-01-13

**Authors:** Wei Cai, Stephanie Yurchak, Diana J. Cole, Laura L. E. Cowen

**Affiliations:** ^1^ Department of Mathematics and Statistics University of Victoria Victoria British Columbia Canada; ^2^ School of Mathematics, Statistics and Actuarial Science University of Kent Canterbury UK

**Keywords:** capture–recapture, identifiability, Jolly‐Seber, parameter redundancy, tag loss

## Abstract

1. Capture–recapture experiments are conducted to estimate population parameters such as population size, survival rates, and capture rates. Typically, individuals are captured and given unique tags, then recaptured over several time periods with the assumption that these tags are not lost. However, for some populations, tag loss cannot be assumed negligible. The Jolly‐Seber tag loss model is used when the no‐tag‐loss assumption is invalid. Further, the model has been extended to incorporate group heterogeneity, which allows parameters to vary by group membership. Many mark–recapture models become overparameterized resulting in the inability to independently estimate parameters. This is known as parameter redundancy.
2. We investigate parameter redundancy using symbolic methods. Because of the complex structure of some tag loss models, the methods cannot always be applied directly. Instead, we develop a simple combination of parameters that can be used to investigate parameter redundancy in tag loss models.
3. The incorporation of tag loss and group heterogeneity into Jolly‐Seber models does not result in further parameter redundancies. Furthermore, using hybrid methods we studied the parameter redundancy caused by data through case studies and generated tag histories with different parameter values.
4. Smaller capture and survival rates are found to cause parameter redundancy in these models. These problems resolve when applied to large populations.

## Graphical abstract

Capture‐recapture experiments where individuals are given unique tags are conducted to estimate population parameters. We investigate the parameter redundancy intrinsic and extrinsic in models that incorporate tag loss. We found that low capture and survival rates can cause extrinsic parameter redundancy.

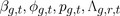

## 1 INTRODUCTION

Mark–recapture studies are often used to estimate population parameters. In open populations, the Jolly‐Seber (JS) model described by Schwarz and Arnason (1996) is widely used. A sample of individuals from the population are marked with a single unique tag, released at an initial stage, and then recaptured at future sample times. Unmarked individuals that are captured can be marked and released at any sample time. As marked and unmarked individuals are assumed to be subject to the same birth/death and immigration/emigration processes, the JS model provides estimates of survival, detection, and entry probabilities. Derived parameter estimates of the number of births and population size can also be obtained. The Jolly‐Seber tag loss model (Cowen & Schwarz, 2006) is based on the JS model. It follows the same assumptions as the JS model but allows for tag loss. Double‐tagging studies have been used to estimate tag loss. A fraction of the individuals are double tagged, and tag loss is assumed to be independent between tags. Individuals that lose all of their tags are either recognized and not retagged, or treated as new individuals. The number of individuals that lose all tags is assumed to be small, with little effect on the parameter estimates. Malcolm‐White et al. (2020) explore this assumption and report on violation conditions.

Double‐tagging or double‐marking studies occur under various study contexts and with various species. There has been a long history of using double tags to estimate tag shedding rates or tag loss probabilities (see Wetherall, 1982; Fabrizio et al., 1999, for discussion). For example, Stevick et al. (2001) used double‐marking of humpback whales to study the use of natural markings in capture–recapture experiments, and Vandergoot et al. (2012) used double tagging to estimate tag loss in Lake Erie walleyes.

Often, group membership (such as males and females) will introduce variability that can bias parameter estimates (Schwarz, 2005, Chap. 8). Xu et al. (2014) developed the group heterogeneity Jolly‐Seber tag loss model (GJSTL), which is an extension of the Jolly‐Seber tag loss (JSTL) model, allowing for parameters to vary by groups.

In the JS model, data are in the form of capture histories, where a 1 or 0 is recorded at each of the *T* sample times to represent if an individual was respectively captured or not. Similarly, tag loss models use tag histories where a 1 or 0 represents the presence or absence of a particular tag. On first capture, 11 is recorded for a double‐tagged individual and 10 for a single‐tagged individual. A traditional capture history may correspond to more than one tag history. For example, a traditional capture history of {1 0 1} for a three sample time experiment could be associated with a tag history of {11 00 11}, {11 00 10}, or {10 00 10} based on whether it has both its tags, or only one tag at each occasion it was captured. The tag history {11 00 10} is an example of tag loss between the first and last sample times. The parameters involved in the probabilities of each tag history for *T* sample times are described below.

*β_g,j_*, the probability that an individual in group *g* enters the population between sample times *j* and *j*+1, *g* = 1, …, *G*; *j* = 0, …, *T*–1. Note $\sum_{j=0}^{T‐1}\beta_{g,j}=1$.

$\Lambda_{g,i,j}$, the probability that an individual in group *g* first tagged at sample time *i* retains its tag between sample times *j* and *j*+1, *g* = 1, …, *G*; *i* = 1, …, *T*–1; *j* = *i*, …, *T*–1.

*p_g,j_*, the probability that an individual in group *g* is captured at sample time *j* given it is alive, *g* = 1, … , *G*; *j* = 1, …, *T*.

*ϕ_g,j_*, the probability that an individual in group *g* survives and remains in the population between sample times *j* and *j*+1 given it is alive at sample time *j*, *g* = 1, …, *G*; *j* = 1, …, *T*–1.

The tag retention parameter, $\Lambda_{g,i,j}$, is the only parameter that is not in the JS model. It appears when an individual is first captured and tagged. It is similar to the survival parameters as it is defined between sample times. All of the parameters can be either time and/or group varying. Tag retention can vary by release group (or cohort). Models can be simplified with parameters constant over time and group for example. We will refer to various models using notation similar to Lebreton et al. (1992). The notation involves a list of parameters with subscripts referring to time dependent, *t*, or group dependent, *g*; no subscript refers to a constant parameter. For example, model $\beta_{t},\phi_{g},p_{g,t},\Lambda$ refers to the model where entry probability *β* varies by time, survival probability ϕ varies by group, capture probability *p* varies over group and time, and tag retention probability Λ is constant.

The super‐population size *N* is captured by modeling the {00 00 ⋯ 00} tag history (Cowen & Schwarz, 2006) and extending to groups requires a super‐population *N_g_* for each group *g*, but ultimately does not add to the parameter list considered in redundancy investigations.

The probability of a tag history, *h*, has components that model the capture, survival, and tag loss processes. For example, the full time varying probability of tag history {11 00 10} for an individual from group 1 would be $\beta_{1,0}p_{1,1}\phi_{1,1}\left\{2\Lambda_{1,1,1}\left(1‐\Lambda_{1,1,1}\right)\right\}\left(1‐p_{1,2}\right)\phi_{1,2}\Lambda_{1,1,2}p_{1,3}+\beta_{1,0}p_{1,1}\phi_{1,1}\Lambda_{1,1,1}^{2}\left(1‐p_{1,2}\right)\phi_{1,2}\left\{2\Lambda_{1,1,2}\left(1‐\Lambda_{1,1,2}\right)\right\}p_{1,3}.$ The first component models the tag loss between sample times 1 and 2. The second component models the possibility of tag loss between sample times 2 and 3.

We investigated the intrinsic parameter redundancy associated with the additional tag loss parameters in Jolly‐Seber type models. We also explored parameter redundancy issues that arise from the data (extrinsic) through generated data sets and case studies. These novel parameter redundancy results will add to the body of knowledge for tag loss models.

## 2 METHODS

### 2.1 Symbolic algorithm

It may be intangible to estimate all of the parameters for certain models. Such a model is called parameter redundant or the parameters are described as non‐identifiable. Structural parameter redundancy is caused by confounded parameters that always appear together in the model in a particular combination, whereas parameter redundancy due to the sparsity in a particular data set is often referred to as estimability. We used the symbolic algebra method (Cole et al., 2010) to test all possible constraints of the GJSTL model for parameter redundancy.

The first stage of the symbolic method involves creating a unique representation of the model known as an exhaustive summary. An exhaustive summary **K**(**θ**) is a vector of parameters that uniquely define the model; **θ** is a vector of all parameters in the model (Cole et al., 2010). In this case, the exhaustive summary is a vector of parameter combinations that uniquely represents the likelihood. As the likelihood is formed from the probabilities of each tag history of the GJSTL model, these form an exhaustive summary. The second stage of the symbolic method is to form a derivative matrix **D** by differentiating the exhaustive summary with respect to each unknown parameter giving D=∂K(θ)/∂θ. The rank of the derivative matrix, **D**, denotes the number of parameters that can be estimated in the model. If the rank of the derivative matrix **D** equals the number of parameters, then the model is not parameter redundant and termed full rank. If the rank is less than the number of parameters then the model is parameter redundant. The deficiency of a model is defined as *d* = *k*–*q*, where *k* is the number of parameters and *q* is the rank of the derivative matrix. A full‐rank model has deficiency zero, and a parameter redundant model has deficiency greater than zero. If a model is parameter redundant, it is possible to determine which of the original parameters are estimable and/or the combinations of the remaining parameters that are estimable. This involves first solving **αD = 0** where **α** denotes the transpose of **α**. This has *d* solutions; the *j*th solution is a vector whose *i*th entry is *α_i,j_*, *i* = 1, …, *k*, *j* = 1, …, *d*. If *α_i,j_* = 0 for all *j*, then the *i*th parameter can still be estimated. Then, a set of *d* partial differential equations ($\sum_{\left(i=1\right)}^{k}\alpha_{\mathrm{ij}}\partial f/\partial \theta_{i}=0,j=1,\cdots ,d,$ where *f* is an arbitrary function) is solved to find other combinations of parameters that can be estimated (Catchpole et al., 1998; Cole et al., 2010).

When models become complex or involve large sample times *T*, the symbolic algebra method becomes computationally infeasible; the rank of the derivative matrix **D** requires large memory space for estimation (see, e.g., Hunter & Caswell, 2009; Jiang et al., 2007). This may be caused by the number of included exhaustive summary terms being too large or too complex. This was the case for complex GJSTL models. In Appendix A, we used the methods of Cole et al. (2010) to develop a simpler exhaustive summary and explain its use.

### 2.2 Hybrid symbolic‐numerical algorithm

Since the symbolic algebra method sometimes can be computationally infeasible, Choquet and Cole (2012) proposed a hybrid symbolic‐numerical algorithm to determine whether a model is parameter redundant. This method finds the derivative matrix symbolically and the rank numerically, by evaluating the derivative matrix at 5 random points in the parameter space (five sets of parameter values). The maximum rank of the 5 points becomes the model rank. In this approach, if **D** is not full rank, then we find the numerical estimation of the left null space **α** of **D** by solving **αD** = 0. As with the symbolic method, there will be *d* solutions that are vectors with entries α*_ij_*. If α*_ij_* is close to zero for all *j*, then the *i*th parameter can still be estimated. However, it is not possible to find estimable parameter combinations (Choquet & Cole, 2012).

This algorithm was adopted for the extrinsic redundancy study in section 3.2; multiple sets of parameter values were applied, which made it difficult to compute the rank and the deficiency for the tested models. An example of this method is in Appendix A (Example 3). Since we are investigating parameter redundancy caused by the data, we do not require the possible reparameterization for confounded parameters.

## 3 RESULTS

### 3.1 Intrinsic parameter redundancy

Parameter redundancy caused by the model is known as intrinsic parameter redundancy. We examined various constraints of the JSTL and the GJSTL models using symbolic algebra, the extension theorem, and the reparameterization method in Maple by applying the simpler exhaustive summaries (Appendix A: Table A2). The constraints included group (*g*), sample times (*t*), and cohort (first tagging time, *r*). We explored all combinations of survival, capture, and entry rates that could vary over group and/or time and tag retention rates that could vary over group, cohort, and/or time. Table 1 lists the parameter redundant models and the full table of parameter redundancy results are given in the Appendix A (Table A4). Among the models examined, only a few were found to be parameter redundant for the same choice of Λ. For example, models $\left\{\beta_{g,t},\phi_{t},p_{g,t},\Lambda \right\}$; $\left\{\beta_{g,t},\phi_{t},p_{g,t},\Lambda_{g}\right\}$; $\left\{\beta_{g,t},\phi_{t},p_{g,t},\Lambda_{t}\right\}$; $\left\{\beta_{g,t},\phi_{t},p_{g,t},\Lambda_{r}\right\}$; $\left\{\beta_{g,t},\phi_{t},p_{g,t},\Lambda_{g,r}\right\}$; $\left\{\beta_{g,t},\phi_{t},p_{g,t},\Lambda_{g,t}\right\}$; $\left\{\beta_{g,t},\phi_{t},p_{g,t},\Lambda_{r,t}\right\}$; and $\left\{\beta_{g,t},\phi_{t},p_{g,t},\Lambda_{g,r,t}\right\}$ all had deficiency 1.

**Table 1:** Parameter redundant GJSTL models which have deficiency greater than 0. The rank gives the number of estimable parameters, and the deficiency is the number of parameters minus the rank. The deficiency (but not the rank) is identical for models where Λ varies over group, cohort, and/or time. The full list of parameter redundancy results is given in Table A4 of Appendix A

Model	Rank	Deficiency
$\beta_{t},\phi_{t},p_{t},\Lambda$	3T‐2	1
$\beta_{t},\phi_{t},p_{t},\Lambda_{g}$	3T+G‐3	1
$\beta_{t},\phi_{t},p_{t},\Lambda_{t}$	4(T‐1)	1
$\beta_{t},\phi_{t},p_{t},\Lambda_{r}$	4(T‐1)	1
$\beta_{t},\phi_{t},p_{t},\Lambda_{g,t}$	5(T‐1)	1
$\beta_{t},\phi_{t},p_{t},\Lambda_{g,r}$	3T‐2+G(T‐1)	1
$\beta_{t},\phi_{t},p_{t},\Lambda_{r,t}$	$3\left(T‐1\right)+\sum_{i=1}^{T‐1}i$	1
$\beta_{t},\phi_{t},p_{t},\Lambda_{g,r,t}$	$3\left(T‐1\right)+G\sum_{i=1}^{T‐1}i$	1
$\beta_{t},\phi_{t},p_{g,t},\Lambda$	T(G+2)‐2	1
$\beta_{t},\phi_{t},p_{g,t},\Lambda_{g}$	T(G+2)+G‐3	1
$\beta_{t},\phi_{t},p_{g,t},\Lambda_{t}$	T(G+3)‐4	1
$\beta_{t},\phi_{t},p_{g,t},\Lambda_{r}$	T(G+3)‐4	1
$\beta_{t},\phi_{t},p_{g,t},\Lambda_{g,t}$	T(G+4)‐5	1
$\beta_{t},\phi_{t},p_{g,t},\Lambda_{g,r}$	T(G+2)‐2+G(T‐1)	1
$\beta_{t},\phi_{t},p_{g,t},\Lambda_{r,t}$	$T\left(G+2\right)‐3+\sum_{i=1}^{T‐1}i$	1
$\beta_{t},\phi_{t},p_{g,t},\Lambda_{g,r,t}$	$T\left(G+2\right)‐3+G\sum_{i=1}^{T‐1}i$	1
$\beta_{g,t},\phi_{t},p_{g,t},\Lambda$	T(2G+1)‐(G+1)	1
$\beta_{g,t},\phi_{t},p_{g,t},\Lambda_{g}$	T(2G+1)‐2	1
$\beta_{g,t},\phi_{t},p_{g,t},\Lambda_{t}$	T(2G+2)‐(G+3)	1
$\beta_{g,t},\phi_{t},p_{g,t},\Lambda_{r}$	T(2G+2)‐(G+3)	1
$\beta_{g,t},\phi_{t},p_{g,t},\Lambda_{g,t}$	T(2G+3)‐(G+4)	1
$\beta_{g,t},\phi_{t},p_{g,t},\Lambda_{g,r}$	T(2G+1)‐(G+1)+G(T‐1)	1
$\beta_{g,t},\phi_{t},p_{g,t},\Lambda_{r,t}$	$T\left(2G+1\right)‐\left(G+2\right)+\sum_{i=1}^{T‐1}i$	1
$\beta_{g,t},\phi_{t},p_{g,t},\Lambda_{g,r,t}$	$T\left(2G+1\right)‐\left(G+2\right)+G\sum_{i=1}^{T‐1}i$	1
$\beta_{g,t},\phi_{g,t},p_{g,t},\Lambda$	3GT‐(2G+1)	*G*
$\beta_{g,t},\phi_{g,t},p_{g,t},\Lambda_{g}$	T(3G)‐(G+2)	*G*
$\beta_{g,t},\phi_{g,t},p_{g,t},\Lambda_{t}$	3(TG‐1)‐2G+T	*G*
$\beta_{g,t},\phi_{g,t},p_{g,t},\Lambda_{r}$	T(3G+1)‐(2G+3)	*G*
$\beta_{g,t},\phi_{g,t},p_{g,t},\Lambda_{g,t}$	3(TG‐1)‐2G+2T‐1	*G*
$\beta_{g,t},\phi_{g,t},p_{g,t},\Lambda_{g,r}$	3GT‐(2G+1)+G(T‐1)	*G*
$\beta_{g,t},\phi_{g,t},p_{g,t},\Lambda_{r,t}$	$3GT‐\left(2G+2\right)+\sum_{i=1}^{T‐1}i$	*G*
$\beta_{g,t},\phi_{g,t},p_{g,t},\Lambda_{g,r,t}$	$3GT‐\left(2G+2\right)+G\sum_{i=1}^{T‐1}i$	*G*

We further explored parameter redundancy in the group heterogeneity tag loss models in terms of how parameters were confounded. We found that group heterogeneity replicates the confounded parameters for each group. For instance when parameters vary with time, one combination of confounded parameters is *c_1_* = *β*_0_ *p*_1_. This same confounding appears in each group with *c*_*g*,1_ = *β*_*g*,0_ *p*_*g*,1_ for *g* = 1, ⋯ , *G*. The full set of confounded parameters for the GJSTL models with parameters varying over group and time is $c_{g,1}=\beta_{g,0}p_{g,1},c_{g,2}=\beta_{g,0}\phi_{g,1}+\beta_{g,1},c_{g,3}=p_{g,T}(1‐\sum_{0}^{\left(T‐2\right)}\beta_{g,i}),c_{g,4}=\phi_{g,T‐1}/(1‐\sum_{0}^{\left(T‐2\right)}\beta_{g,i}),$ where groups *g* = 1, …, *G*. The confounded parameters for the JSTL models with parameters varying over time are the same but without the groups (Appendix A: Table A4).

Schwarz et al. (1993) reported a list of confounded parameters for the JS model, but did not discuss their method for obtaining these confounded parameters. It is possible to show that the confounded parameters *c_g_*_,1,_ *c_g_*_,2,_ *c_g_*_,3,_ and *c_g_*_,4_ are equivalent in form to the ones reported in Schwarz et al. (1993). By letting $\beta_{g,T‐1}=\left(1‐\sum_{0}^{T‐2}\beta_{g,i}\right)$ and with simple algebra and rearrangement, the confounded parameters become $\beta_{g,0}p_{g,1},\beta_{g,T‐1}/\phi_{g,T‐1}=1/c_{g,4},\phi_{g,T‐1}p_{g,t}=c_{g,3}\times c_{g,4}$, and $\beta_{g,1}+\beta_{g,0}\left(1‐p_{g,1}\right)\phi_{g,1}=c_{g,2}‐c_{g,1}\phi_{g,1}$. This is identical to the confounded parameters given in Schwarz et al. (1993) with the addition of groups. Thus, extending the JS model with tag loss and groups does not affect what can be estimated within this model.

Additive models present another class of models to consider. An additive constraint across group may allow for independent estimation of parameters (Viallefont et al., 1998); however, where the additive constraint is placed is important. Gimenez et al. (2003) describe models with additive effects; standard parameters such as *p_t,g_* are expressed as some function *f*(*a_t_*, *m*) where *a_t_* would be parameters for time effects and *m*a parameter for group effects. Assuming two groups, the choice of *f*(*a_t_*, *m*) could be either a logit‐scale where $p_{1,t}=\exp\left(a_{t}+m\right)/\left[1+\exp\left(a_{t}+m\right)\right]$ for group 1 and $p_{2,1}=exp(a_{t})/[1+exp(a_{t})]$ for group 2, or a log‐scale where *p*_1,*t*_ = exp(*a_t_*+*m*) for group 1 and *p*_2,1_ = exp(*a_t_*) for group 2. Results in Gimenez et al. (2003) and Viallefont (1995) erroneously suggest that the additive model could resolve issues with parameter redundancies in the Cormack‐Jolly‐Seber model, due to an analytic computation error in older versions of Maple™. Choquet and Cole (2012) found that the additive model did not reduce the deficiency.

We considered including additive effects to the group and time dependent model, *β_g,t_*, *ϕ_g,t_*, *p_g,t_*, $\Lambda_{g,t}$, with two groups. This model has deficiency 2. Adding either a logit‐scale or log‐scale additive effect to *p_g,t_* reduces the deficiency to 0, whereas adding either a logit‐scale or log‐scale additive effect to *ϕ_g,t_* reduces the deficiency to 1. The additive effect can reduce the deficiency; however, it has to be on *p_g,t_* to give an identifiable model.

### 3.2 Extrinsic parameter redundancy

Extrinsic parameter redundancy is caused by the data (Gimenez et al., 2004) and is often referred to as estimability (Lele et al., 2010). Here, we used the expected data set approach of Cole et al. (2014). When considering extrinsic parameter redundancy, only the presence of a tag history matters, rather than tag history frequency. That is, the parameter redundancy would be the same if any specific tag history appeared just once or 100 times. However, redundancy may change if the tag history is not present. Therefore, we considered whether we expected a history to be present or not. For specific parameter values, the probability *P*(*h*) of the occurrence of each tag history *h* can be found. Suppose that *n* animals are tagged in each group, then the number of individuals we expect to see is *E*(*h*) =*n*× *P*(*h*). An exhaustive summary was created with terms corresponding to the probability *P*(*h*) of the occurrence of each tag history *h*. A tag history was included if *E*(*h*) ≥ 1, meaning we expected to see at least one individual with history *h*. Any tag history with *E*(*h*) < 1 was excluded.

We used the following parameter values to generate tag histories. Note that the fraction of double‐tagged individuals among the tagged individuals was the same (0.3) in all cases. The entry rates were 1/*T*. The capture rates *p* were set to 0.1, 0.5, and 0.8. The survival rates ϕ were 0.2, 0.5, and 0.8.b To accommodate tag technology improvements, we set a wide range of parameter values for the survival probability with consideration for future studies of any species that might be double tagged (not only long lived species). Tag retention rates Λ were motivated by the literature. We found tag retention rates to vary depending on many factors such as the species, age, tag types, tagging season, and experimental conditions. The tag retention rates ranged from 13% (Fogarty & Russell, 1980) to 95% (Gonzalez‐Vicente et al., 2012) for lobsters only, depending on different experimental methods. Adelie penguins had high tag retention rates 88% to 100% depending on band ages (Ainley & DeMaster, 1980). Estimates for double‐tagging experiments also have a wide range. For instance, Pistorius et al. (2000) conducted an experiment on elephant seals where the tag retention rate of retaining both tags for males was 65% while that of females was 83%. The values of the tag retention rates Λ we used were 0.2, 0.5, and 0.8. The combination of the three values for each parameter gave 27 tag history sets.

We generated tag histories for the 27 sets of parameter values for *T* = 3 sample times, *G* = 1 or 2, and *n* = 1000. We excluded the tag history terms with the expected number E(h) < 1. Some data sets were found to have the same set of tag histories, such as tag history sets Λ=0.2,p=0.1,ϕ=0.2 and Λ=0.2,p=0.1,ϕ=0.5. This reduced the number of tag history sets to 18. The hybrid symbolic‐numeric method was used to find the deficiency.

Some sets of parameter values caused an increase in the deficiency compared to the intrinsic parameter redundancy results of Section 3.1. This inconsistency occurred because a key exhaustive summary term was missing due to the data. The sets causing an increase in the deficiency were mainly the ones with smaller parameter values.

Example 1

Table 2 shows an example of extrinsic parameter redundancy results for two groups with *n* = 1000 and parameter values of Λ=0.2,p=0.1,ϕ=0.2 were used. When all tag histories are included, the deficiency for all the models with $\beta_{g,t},\phi_{g,t},p_{g,t}$ was 2 regardless of the constraint on Λ. However, for the set of tag histories with Λ=0.2,p=0.1,ϕ=0.2 the deficiency was larger and changed with the constraint on Λ as crucial tag histories were unobserved. This is different from our conclusion in Section 3.1 that the addition of tag retention rate does not affect the deficiency.

**Table 2:** Evaluation of $\beta_{g,t},\phi_{g,t},p_{g,t},\Lambda_{x}$ models with tag history generated using entry rate β=1/T, tag retention rate Λ=0.2, capture rate p=0.1, survival rate ϕ=0.2, *n* = 1000, *G* = 2. Here, *r* denotes varying by cohort. Note that for data with all possible tag histories the deficiency would be *G* = 2 (see Appendix A: Table A4)

Models	Rank	Deficiency
$\beta_{g,t},\phi_{g,t},p_{g,t},\Lambda$	4*T* ‐ 2	2*T* ‐ 1
$\beta_{g,t},\phi_{g,t},p_{g,t},\Lambda_{g}$	4*T* ‐ 2	2*T*
$\beta_{g,t},\phi_{g,t},p_{g,t},\Lambda_{r}$	4*T* ‐ 2	4*T* ‐ 6
$\beta_{g,t},\phi_{g,t},p_{g,t},\Lambda_{t}$	4*T* ‐ 2	3*T* ‐ 3
$\beta_{g,t},\phi_{g,t},p_{g,t},\Lambda_{g,r}$	4*T* ‐ 2	4*T* ‐ 4
$\beta_{g,t},\phi_{g,t},p_{g,t},\Lambda_{g,t}$	4*T* ‐ 2	5*T* ‐ 7
$\beta_{g,t},\phi_{g,t},p_{g,t},\Lambda_{r,t}$	4*T* ‐ 2	5*T* ‐ 8
$\beta_{g,t},\phi_{g,t},p_{g,t},\Lambda_{g,r,t}$	4*T* ‐ 2	8*T* ‐ 14

To determine the effect of the number of animals tagged, we increased *n* to 10,000, 100,000, and 1,000,000 for the sets that had data‐driven changes in the deficiency of a model. These models are listed in Table 3, alongside how large *n* must be before parameter redundancy results are identical to when all the tag histories are present. It turned out that increasing *n* removed the additional parameter redundancy caused by the data. If *n* increases from 1,000 to 10,000, many of the additional deficiencies disappear. However, for the Λ=0.2, *p* = 0.1, ϕ = 0.2 case, the additional deficiency did not disappear until *n* = 1,000,000 (Table 3). Therefore, data‐driven parameter redundancy in the GJSTL models disappear when there is a large *n*. We note that in this case of low survival and capture rates, there are large biases in parameter estimates (Malcolm‐White et al., 2020); these biases are somewhat improved by high tag retention rates.

**Table 3:** Number of tagged animals *n* required for the extrinsic parameter redundancy results to be identical to intrinsic parameter redundancy results

*n*	Parameter Value Combination
1,000,000	Λ=0.2,p=0.1,ϕ=0.2
100,000	Λ=0.2,p=0.5,ϕ=0.2
10,000	Λ=0.2,p=0.5,ϕ=0.8
	Λ=0.5,p=0.1,ϕ=0.8
	Λ=0.5,p=0.5,ϕ=0.2
	Λ=0.9,p=0.1,ϕ=0.8
	Λ=0.9,p=0.5,ϕ=0.2
	Λ=0.9,p=0.8,ϕ=0.5

## 4 CASE STUDIES

Southern rock lobster *Jasus edwardsii* are commercially fished in Tasmania, Australia, and studied through a long‐term tagging program. There were 5,993 females and 5,514 males single or double tagged upon initial capture and followed for 8 years from 1999–2006. Xu et al. (2014) applied the GJSTL model to these data (where sex determined group membership) modifying it slightly to have annual sampling occasions. They used AIC for model selection which chose model $b_{t}^{⋆},\phi_{g},p_{g,t},\Lambda_{g,t}$. However, Xu et al. noted that they avoided fitting entry probabilities that varied by group so as to avoid parameter redundant models. The parameter b^*^_g,j_ is a function of the parameter *β_g,j_* and interpreted as the expected fraction of the population in group *g* remaining to enter the population that enters between sample times *j* and *j*+1. It maps to *β_g,j_* using the function

$$b_{g,j}^{⋆}=\left\{\begin{array}{cc} & \beta_{g,0}if\;j=0;\\ & \frac{\beta_{g,j}}{\sum_{u=j}^{T‐1}\beta_{g,u}}if\;j=1,\cdots ,T‐2;\\ & 1\;if\;j=T‐1. \end{array}\right)$$

To investigate extrinsic parameter redundancy (cause by the data), we considered the 11 models with highest AIC from Xu et al. (2014), compared to the intrinsic parameter redundancy results (caused by the model) (Table 4). We found that the extrinsic deficiency was identical to the intrinsic deficiency. This was largely due to the large sample size that allowed for a wide variety of tag histories allowing for parameter identifiability.

**Table 4:** Comparison of intrinsic and extrinsic parameter deficiencies in the southern rock lobster data 1999–2006 and the walleye data 2009–2011

	Intrinsic	Extrinsic deficiency	
Model	Deficiency	Lobster	Walleye
$\beta_{t},\;\phi_{g},p_{g,t},\;\Lambda_{g,t}$	0	0	3
$\beta_{t},\phi_{g,t},p_{g,t},\;\Lambda_{g,t}$	0	0	5
$\beta_{t},\phi_{g},p_{g,t},\;\Lambda_{g}$	0	0	3
$\beta_{t},\phi_{t},p_{t},\;\Lambda_{t}$	1	1	2
$\beta_{t},\phi_{g,t},p_{g},\;\Lambda_{g,t}$	0	0	1
$\beta_{t},\phi_{g,t},p_{g},\;\Lambda_{t}$	0	0	1
$\beta_{t},\phi_{g},p_{g},\;\Lambda_{g}$	0	0	0
$\beta_{t},\phi_{g},p_{g},\;\Lambda$	0	0	0
$\beta_{t},\phi_{g},p,\;\Lambda_{g}$	0	0	0
$\beta_{t},\phi ,p,\;\Lambda$	0	0	0
$\beta_{t},\phi_{g},p,\;\Lambda$	0	0	0

We also considered a much smaller mark–recapture data set of walleye *Sander vitreus*. The study was conducted in the Woman Chain of lakes in northern Cass County, Minnesota. Fish were tagged with t‐bar anchor tags a week or two each April to early May as they entered the Boy River to spawn between 2009 and 2011. They were given single or double tags resulting in 1,108 females and 1,473 males tagged over the 3‐year period.

For comparison, we used the same 11 models as the lobster data set. More models were considered beyond these 11 models with results in the Appendix A: Table A5. These data did affect parameter redundancy results, with increased deficiency for some of the models (Table 4). The change in deficiency occurred when certain tag histories were unobserved. In this case, there were many missing histories, including those for individuals captured for the first time in 2011 (tag histories {00 00 11} or {00 00 01}). Key tag histories are more likely to be missing when there is a small sample size or for sets of parameters with low values.

## 5 DISCUSSION

We investigated the parameter redundancy caused by model structure and data in Jolly‐Seber tag loss models, with a focus on various constraints on JSTL and GJSTL models. We used the symbolic algebra method to determine the confounded parameters, which were found to be the same for JSTL and JS models, showing that the addition of independent tag retention parameters does not result in any further structural redundancies. The incorporation of group heterogeneity only replicates the confounded parameters of the JSTL models for each group, with no additional confounded parameters.

Although the symbolic algebra method is effective to assess parameter redundancy for JSTL and GSTL models, we found that it had large memory requirements for some complex models. Hence, the hybrid symbolic‐numerical algorithm can be used to investigate parameter redundancies caused by data.

We note that an increase in deficiency due to data is eliminated by increasing the number of individuals tagged. For many parameter value combinations, there were no data‐related redundancy increases, even with *n* = 1000. However, for one set of parameter values we had to increase *n* to 1,000,000 before there was no increase in data‐related deficiency. We recommend for scenarios with low capture probability, low survival probability, and/or low tag retention probability and small *n* (<1,000), extrinsic parameter redundancy should be investigated per specific data set. Otherwise, the general intrinsic parameter redundancy results in Table 1 and Appendix A: Table A4 hold.

The walleye case study exhibited extrinsic parameter redundancy due to sparse data. This occurred because some tag histories were not included in the data. When conducting a capture–recapture study involving tag loss, we recommend the study be designed to obtain as many tag histories as possible. The walleye study did not include capturing and tagging individuals at the last sample time, and this caused some of the extrinsic parameter redundancy.

Future plans include assessment of parameter redundancy for models where tag loss is dependent. The independence assumption for tag loss is limiting as this assumption has been clearly violated in several contexts, such as southern elephant seals (see Schwarz et al., 2012). We are currently developing a model for dependent tag loss. Further, incorporating covariates into these models is expected to also decrease redundancies (see Cole & Morgan, 2010). This should be examined within individual studies.

## CONFLICT OF INTERESTS

None declared.

## AUTHOR CONTRIBUTION

Wei Cai: Investigation (equal); Writing‐original draft (supporting); Writing‐review and editing (supporting). Stephanie Yurchak: Formal analysis (equal); Investigation (equal); Writing‐original draft (lead); Writing‐review and editing (supporting). Diana Cole: Formal analysis (supporting); Methodology (lead); Software (lead); Writing‐original draft (supporting); Writing‐review and editing (supporting). Laura Cowen: Conceptualization (lead); Formal analysis (supporting); Funding acquisition (lead); Investigation (supporting); Methodology (supporting); Project administration (lead); Resources (lead); Supervision (lead); Writing‐original draft (supporting); Writing‐review and editing (supporting).

## Data Availability

Data, R scripts, and Matlab scripts are available from the Dryad Digital Repository https://doi.org/10.5061/dryad.7wm37pvrn

## Appendix A

### ADDITIONAL METHODS

#### Simpler exhaustive summary

As discussed in the main paper, for complex GJSTL models it is computationally infeasible to calculate the rank of the derivative matrix. It is necessary to create a simpler exhaustive summary (**s**) for the models of this paper. We can find a simpler exhaustive summary for **K**(**θ**) by first proposing a reparameterization **s**(**θ**). We then rewrite **K**(**θ**) as a function of ***s***, **K**(**s**). If the derivative matrix ∂K(s)/∂s is full rank, then **s** is an exhaustive summary. If it is not full rank, then a new parameterization can be found by solving an appropriate set of partial differential equations. If ∂s(θ)/∂θ is also full rank, then the rank of ∂K(s)/∂s will be the same as the rank of ∂K(θ)/∂θ. This is the reparameterization theorem from Cole et al. (2010). For example, this method has been used to create simpler exhaustive summaries in multi‐state models (Cole et al., 2012) and multi‐event models (Cole et al., 2014).

Here, the original exhaustive summary, **K**(*θ*), is a vector containing the probabilities of each tag history. We illustrate this method in Example 1 below.

Example 1

Consider the time dependent JSTL model with sample times *T* = 2. The probabilities for each tag history are given in Table A1. The terms in Table A1 form the exhaustive summary **K**(*θ*) with *θ* = [$\beta_{0},\phi_{1},p_{1},p_{2},\Lambda_{1,1}$]. The estimable parameters are found to be $\beta_{0}p_{1},\phi_{1}p_{2},\left\{1+\beta_{0}\left(\phi_{1}‐1\right)\right\}/\phi_{1}$ and $\Lambda_{1,1}$. A reparameterization of **K**(*θ*) is *s* = [*s*_1,_ *s*_2,_ *s*_3,_ *s*_4_] =$\beta_{0}p_{1},\phi_{1}p_{2},\left\{\beta_{0}\phi_{1}\left(1‐p_{1}\right)+1‐\beta_{0}\right\}p_{2},\Lambda_{1,1}$ $\left[\beta_{0}p_{1},\phi_{1}p_{2},\left\{\beta_{0}\phi_{1}\left(1‐p_{1}\right)+1‐\beta_{0}\right\}p_{2},\Lambda_{1,1}\right]$, where s_3_ is the probability of tag history 0011 and 0010 and is found by manipulating the estimable parameters. Rewriting **K**(**θ**) in terms of *s* gives **K**(**s**) = $s_{1}s_{2}s_{4},s_{1}s_{2}s_{4}^{2},s_{1}s_{2}s_{4}\left(1‐s_{4}\right),s_{1}\left(1‐s_{2}s_{4}\right),s_{1}\left(1‐2s_{2}s_{4}+s_{2}s_{4}^{2}\right),s_{3}]$.

The derivative matrix of **K(s)** with respect to **s** is full rank, which implies that **s** is a simpler exhaustive summary for the time‐dependent JSTL model with sample times *T* = 2.

In general, the terms needed for a simpler exhaustive summary, **K*_T_*** (**θ**_T_) for the time dependent JSTL model with *T* sample times are given in Table A2. If groups are included in the model, the simpler exhaustive summary is repeated for each group.

The simpler exhaustive summary, **K*_T_*** (**θ**_T_), is not necessarily valid for every possible constraint on the parameters. We can check whether any nested models are parameter redundant within a full‐rank model using a PLUR decomposition of the derivative matrix and examining the determinant of one of the resulting matrices (Cole et al., 2010). The same procedure applies for checking the exhaustive summary. We write the derivative matrix, $D_{T}=\partial K_{T}\left(\theta_{T}\right)/\partial \theta_{T}$, as a product of 4 matrices, **P**, a permutation matrix consisting of 0s and 1s, **L**, a lower diagonal matrix with 1s on the diagonal, **U**, an upper diagonal matrix with any entry on the diagonal, and **R**, a matrix in reduced echelon form. If Det (**U**) = 0 for any constraints, then **K_*T*_** (**θ**_T_) will not be an exhaustive summary for the constrained model.

#### Extension theorem

The extension theorem was introduced by Catchpole and Morgan (1997) and generalized in Cole et al. (2010). Suppose a full‐rank model with exhaustive summary **K** *_p_*_1_ and parameters *θ*_*p*1_ has a derivative matrix $D_{p1}\;=\;\partial K_{p1}\left(\theta_{p1}\right)/\partial \theta_{p1}$. By adding extra exhaustive terms **K** *_p_*_2_ and extra parameters *θ*_*p*2_, the model is extended. Construct a derivative matrix **D** *_p_*_2_ by differentiating the extra exhaustive summary terms with respect to the extra parameters $D_{p2}\;=\;\partial K_{p2}\left(\theta_{p2}\right)/\partial \theta_{p2}$. Then, the derivative matrix for the extended model is

$$D=\left(\begin{array}{cc} D_{p1} & \frac{\partial K_{p2}\left(\theta_{p1}\right)}{\partial \theta_{p1}}\\ 0 & D_{p2} \end{array}\right)$$

The extension theorem states that if the derivative matrices **D** *_p_*_1_ and **D** *_p_*_2_ are full rank then the extended model is full rank. This is trivially true if the number of extra parameters is zero or one. Results can be generalized for any sample time *T* by induction extension theorem (Catchpole & Morgan, 1997).

The extension theorem is demonstrated in Example 2 below.

Example 2

The model $\beta_{t},\phi_{t},p,\Lambda$ with *T* = 3 sample times has a simpler exhaustive summary

$$K=\left[\begin{array}{c} \phi_{1}p\\ \phi_{2}p\\ \left(1‐p\right)\phi_{1}\\ \Lambda \\ \Lambda^{2}\\ \Lambda \left(1‐\Lambda \right)\\ \beta_{0}p\\ \left\{\beta_{0}\left(1‐p\right)\phi_{1}+\beta_{1}\right\}p\\ \{\beta_{0}{\left(1‐p\right)}^{2}\phi_{1}\phi_{2}+\beta_{1}\left(1‐p\right)\phi_{2}+1‐\beta_{0}‐\beta_{1}\}p \end{array}\right]$$

and parameters $\theta \;=\;\left[p,\Lambda ,\phi_{1},\phi_{2},\beta_{0},\beta_{1}\right]$. The derivative matrix ∂K/∂θ has full rank 6.

Extending the model to *T* = 4 sample times requires an adjustment to the extension theorem because 1–*β*_0_–*β*_1_ is replaced by *β*_2_. This is known as the two‐stage extension theorem (Hubbard et al., 2014). Part 1 consists of the terms

$$K_{p1}=\left[\begin{array}{c} \phi_{1}p\\ \phi_{2}p\\ \left(1‐p\right)\phi_{1}\\ \Lambda \\ \Lambda^{2}\\ \Lambda \left(1‐\Lambda \right)\\ \beta_{0}p\\ \left\{\beta_{0}\left(1‐p\right)\phi_{1}+\beta_{1}\right\}p \end{array}\right]$$

and parameters $\theta_{p1}\;=\;\left[p,\Lambda ,\phi_{1},\phi_{2},\beta_{0},\beta_{1}\right]$. The derivative matrix $\partial K_{p1}\;/\;\partial \theta_{p1}$ has full rank 6 as well.

The second part consists of the terms

$$K_{p2}=\left[\begin{array}{c} \phi_{3}p\\ \left(1‐p\right)\phi_{2}\\ \Lambda^{3}\\ \Lambda^{2}\left(1‐\Lambda \right)\\ \{\beta_{0}{\left(1‐p\right)}^{2}\phi_{1}\phi_{2}+\beta_{1}\left(1‐p\right)\phi_{2}+\beta_{2}\}p\\ \{\beta_{0}{\left(1‐p\right)}^{3}\phi_{1}\phi_{2}\phi_{3}+\beta_{1}{\left(1‐p\right)}^{2}\phi_{2}\phi_{3}+\beta_{2}\left(1‐p\right)\phi_{3}+1‐\beta_{1}‐\beta_{2}‐\beta_{3}\}p \end{array}\right]$$

with extra parameters $\theta_{p2}\;=\;\left[\phi_{3},\beta_{2}\right]$. The derivative matrix $\partial K_{p2}\;/\;\partial \theta_{p2}$ has full rank 2. Since **D** *_p_*_1_ and **D** *_p_*_2_ are both full rank, it follows from the extension theorem that the extended, four sample time model is full rank. When this model is extended from *T*=*t* to *t*+1 sample times, the extra parameters added are *ϕ_t_* and $\beta_{t‐1}$. The extra exhaustive summary terms will be similar to those in **K** *_p_*_2_. If the *t* sample time model is full rank, then the *t*+1 sample time model will be full rank from the extension theorem. By induction, it can be concluded that this model will be full rank for any sample time *T*.

If the model is parameter redundant, we combine the reparameterization and extension theorems to obtain general results Cole et al. (2010) and Hubbard et al. (2014).

Example 3

Consider the model $\beta_{t},\phi_{t},p_{t},\Lambda$ where tag retention is constant and all the other parameters are time dependent. An exhaustive summary for the three sample time model is

$$K_{3}=\left[\begin{array}{c} \left\{\beta_{0}\left(1‐p_{1}\right)\phi_{1}+\beta_{1}\right\}p_{2}\\ \phi_{2}p_{3}\\ \Lambda \\ \left\{\beta_{0}\left(1‐p_{1}\right)\left(1‐p_{2}\right)\phi_{1}\phi_{2}+\beta_{1}\left(1‐p_{2}\right)\phi_{2}+1‐\beta_{0}‐\beta_{1}\right\}p_{3}\\ \beta_{0}p_{1}\\ \phi_{1}p_{2}\\ \left(1‐p_{2}\right)\phi_{1}\\ \Lambda^{2}\\ \Lambda \left(1‐\Lambda \right) \end{array}\right]$$

The derivative matrix formed from differentiating **K**_3_ with respect to the parameters $\theta_{3}=\left[\beta_{0},\beta_{1},\phi_{1},\phi_{2},p_{1},p_{2},p_{3},\Lambda \right]$ has rank 7, but there are eight parameters. Therefore to use the extension theorem, we first need to reparameterize to seven parameters. A reparameterization of the three sample time model is $s_{3}=\left[s_{1},s_{2},s_{3},s_{4},s_{5},s_{6},s_{7}\right]=\left[\Lambda ,p_{2},\phi_{1},\beta_{0}p_{1},\beta_{0}\phi_{1}+\beta_{1},p_{3}\left(1‐\beta_{0}‐\beta_{1}\right),\phi_{2}/\left(1‐\beta_{0}‐\beta_{1}\right)\right]$ .

This gives $K_{3}\left(s_{3}\right)={\left[\left(s_{5}‐s_{3}s_{4}\right)s_{2},s_{6}s_{7},s_{1},s_{6}\left\{1+s_{7}\left(1‐s_{2}\right)\left(s_{5}‐s_{3}s_{4}\right)\right\},s_{4},s_{2}s_{3},s_{3}\left(1‐s_{2}\right),s_{1}^{2},s_{1}\left(1‐s_{1}\right)\right]}^{T}$. The derivative matrix $D_{s_{3}}\;=\;\partial K_{3}\left(s_{3}\right)/\partial s_{3}$ has full rank 7. After reparameterizing the four sample time model, the extra exhaustive summary terms used to extend the three to the four sample time model are found to be $K_{\mathrm{ext}}\left(s_{4}\right)={\left[s_{9}s_{10},s_{9}\left\{s_{7}s_{10}\left(1‐s_{2}\right)\left\{s_{3}s_{4}\left(s_{6}‐s_{8}\right)+s_{5}\left(s_{8}‐s_{6}\right)\right\}+s_{10}\left(s_{8}‐s_{6}\right)+1\right\},s_{7}\left(s_{8}‐s_{6}\right),s_{1}^{3},s_{1}^{2}\left(1‐s_{1}\right)\right]}^{\prime }$. The extra parameters are *s_8_ = β_2_, s_8_ = p_4_ (1–β_0_–β_1_–β_2_)* and $s_{10}=\phi_{3}/\left(1‐\beta_{0}‐\beta_{1}‐\beta_{2}\right)$, which gives $s_{\mathrm{ext}}\;=\;\left[s_{8},s_{9},s_{10}\right]$. The derivative matrix $D_{\mathrm{ext}}\;=\;\partial K_{\mathrm{ext}}\left(s_{4}\right)/\partial s_{\mathrm{ext}}$ has full rank 3, so by the extension theorem the reparameterized model has full rank 10. By induction, the reparameterized model will always have full rank 3*T*–2. By the reparameterization theorem, the original model will have rank 3*T*–2. Since the original model has 3*T*–1 parameters, it will always be parameter redundant with deficiency 1.

### ADDITIONAL RESULTS

A full listing of all models investigated for parameter redundancy is shown below.

Table A3 shows the parameter redundancy results for Jolly‐Seber (JS) models. Table A4 shows the parameter redundancy results for group heterogeneity Jolly‐Seber tag loss model (GJSTL) models. The deficiency of each model is given alongside the estimable parameter combinations when the deficiency is greater than zero. A deficiency of zero indicates the model is not parameter redundant. The estimable parameter combinations specify which parameters are identifiable and which parameters are confounded when a model is parameter redundant. In the model notation *t* represents varying by time, *g* represents varying by group, and *r* represents varying by release cohort. A lack of subscript indicates the parameter is constant over time, group, or release cohort. In the estimable parameter combinations, *T* is the number of sample occasions and *G* is the number of groups.

Table A5 shows a more extensive comparison of the extrinsic parameter redundancy in the southern rock lobster vs. walleye data sets for various models.

**Table A1:** Exhaustive summary for the Jolly‐Seber tag loss (JSTL) models. Probabilities of tag histories for a *T* = 2 sample time study. Note that β_1_ = 1–β_0_

Tag history	Probability
10 10	$\beta_{0}\phi_{1}p_{1}p_{2}\Lambda_{1,1}$
11 11	$\beta_{0}\phi_{1}p_{1}p_{2}\Lambda_{1,1}^{2}$
11 10	$\beta_{0}\phi_{1}p_{1}p_{2}\Lambda_{1,1}\left(1‐\Lambda_{1,1}\right)$
10 00	$\beta_{0}p_{1}\left(1‐\phi_{1}+\phi_{1}\left(1‐p_{2}\right)\Lambda_{1,1}+\phi_{1}\left(1‐\Lambda_{1,1}\right)\right)$
11 00	$\beta_{0}p_{1}\left(1‐\phi_{1}+\phi_{1}\left(1‐p_{2}\right)\Lambda_{1,1}^{2}+2\phi_{1}\left(1‐p_{2}\right)\Lambda_{1,1}\left(1‐\Lambda_{1,1}\right)+\phi_{1}{\left(1‐\Lambda_{1},1\right)}^{2}\right)$
00 10 and 00 11	$\left(\beta_{0}\left(1‐p_{1}\right)\phi_{1}+1‐\beta_{0}\right)p_{2}$

**Table A2:** Terms needed in a simpler exhaustive summary for the JSTL model. Note that for the GJSTL model, terms are replicated by group

The probability of	Exhaustive summary terms
First capture at each sample time	$\beta_{0}p_{1},\left(\beta_{0}\left(1‐p_{1}\right)\phi_{1}+\beta_{1}\right)p_{2}$
Surviving and being captured	$\phi_{1}p_{2}$,,$\phi_{T‐1}p_{T}$
Surviving and not being captured	$\phi_{1}\left(1‐p_{2}\right)$,,$\phi_{T‐2}\left(1‐p_{T‐1}\right)$
Retaining tags since first capture	$\Lambda_{1,1}\Lambda_{1,2}\times \cdots \times \Lambda_{1,T‐1}$,$\Lambda_{T‐2,T‐2}\Lambda_{T‐2,T‐1}$,$\Lambda_{T‐1,T‐1}$
Losing one tag since first capture	$\Lambda_{1,1}\left(1‐\Lambda_{1,2}\right)\Lambda_{1,3}\times \cdots \times \Lambda_{1,T‐1}$, $\Lambda_{T‐2,T‐2}\left(1‐\Lambda_{T‐2,T‐1}\right)$

**Table A3:** Deficiency of JS models. A deficiency of 0 indicates the model is not parameter redundant. A deficiency of greater than 0 indicates the number of redundant parameters. When the deficiency is greater than 0, the table also provides the estimable parameter combinations

Model	Deficiency	Estimable Parameter combinations
$\beta_{t},\phi ,p$	0	
$\beta_{t},\phi ,p_{g}$	0	
$\beta_{t},\phi ,p_{t}$	0	
$\beta_{t},\phi ,p_{g,t}$	0	
$\beta_{t},\phi_{g},p$	0	
$\beta_{t},\phi_{g},p_{g}$	0	
$\beta_{t},\phi_{g},p_{t}$	0	
$\beta_{t},\phi_{g},p_{g,t}$	0	
$\beta_{t},\phi_{t},p$	0	
$\beta_{t},\phi_{t},p_{g}$	0	
$\beta_{t},\phi_{t},p_{t}$	1	N, $\phi_{1},\cdots ,\phi_{T‐2}$, $\beta_{2},\cdots ,\beta_{T‐2}$, $p_{2},\cdots ,p_{T‐1}$, $\beta_{0}p_{1}$,
		$\beta_{0}\phi_{1}+\beta_{1}$, $\phi_{T‐1}p_{T}$, $p_{T}\beta_{T‐1}$
$\beta_{t},\phi_{t},p_{g,t}$	1	$N_{g}$, $\phi_{1},\cdots ,\phi_{T‐2}$, $\beta_{2},\cdots ,\beta_{T‐2}$, $p_{g,2},\cdots ,p_{g,T‐1}$,
		$\beta_{0}p_{g,1}$, $\beta_{0}\phi_{1}+\beta_{1}$, $\phi_{T‐1}p_{g,T}$, $\beta_{T‐1}/\phi_{T‐1}$
$\beta_{t},\phi_{g,t},p$	0	
$\beta_{t},\phi_{g,t},p_{g}$	0	
$\beta_{t},\phi_{g,t},p_{t}$	0	
$\beta_{t},\phi_{g,t},p_{g,t}$	0	
$\beta_{g,t},\phi ,p$	0	
$\beta_{g,t},\phi ,p_{g}$	0	
$\beta_{g,t},\phi ,p_{t}$	0	
$\beta_{g,t},\phi ,p_{g,t}$	0	
$\beta_{g,t},\phi_{g},p$	0	
$\beta_{g,t},\phi_{g},p_{g}$	0	
$\beta_{g,t},\phi_{g},p_{t}$	0	
$\beta_{g,t},\phi_{g},p_{g,t}$	0	
$\beta_{g,t},\phi_{t},p$	0	
$\beta_{g,t},\phi_{t},p_{g}$	0	
$\beta_{g,t},\phi_{t},p_{t}$	0	
$\beta_{g,t},\phi_{t},p_{g,t}$	1	$N_{g}$, $\phi_{1},\cdots ,\phi_{T‐2}$, $\beta_{g,2},\cdots ,\beta_{g,T‐2}$, $p_{g,2},\cdots ,p_{g,T‐1}$,
		$\beta_{g,0}p_{g,1}$, $\beta_{g,0}\phi_{1}+\beta_{g,1}$, $p_{g,T}\beta_{g,T‐1}$,
		$\phi_{1,T‐1}p_{1,T},\cdots ,\phi_{G‐1,T‐1}p_{G‐1,T}$, $f(\beta_{g,i},\phi_{1})$
$\beta_{g,t},\phi_{g,t},p$	0	
$\beta_{g,t},\phi_{g,t},p_{g}$	0	
$\beta_{g,t},\phi_{g,t},p_{t}$	0	
$\beta_{g,t},\phi_{g,t},p_{g,t}$	G	$N_{g}$, $\phi_{g,1},\cdots ,\phi_{g,T‐2}$, $\beta_{g,2},\cdots ,\beta_{g,T‐2}$, $p_{g,2},\cdots ,p_{g,T‐1}$,
		$\beta_{g,0}p_{g,1}$, $\beta_{g,0}\phi_{g,1}+\beta_{g,1}$, $\phi_{g,T‐1}p_{g,T}$, $p_{g,T}\beta_{g,T‐1}$

**Table A4:** Deficiency of GJSTL models. A deficiency of 0 indicates the model is not parameter redundant. A deficiency of greater than 0 indicates the number of redundant parameters

Model	Deficiency	Estimable parameter combinations
β,ϕ,p,Λ	0
$\beta ,\phi ,p,\Lambda_{g}$	0
$\beta ,\phi ,p,\Lambda_{r}$	0
$\beta ,\phi ,p,\Lambda_{t}$	0
$\beta ,\phi ,p,\Lambda_{g,r}$	0
$\beta ,\phi ,p,\Lambda_{g,t}$	0
$\beta ,\phi ,p,\Lambda_{r,t}$	0
$\beta ,\phi ,p,\Lambda_{g,r,t}$	0
$\beta ,\phi ,p_{g},\Lambda$	0
$\beta ,\phi ,p_{g},\Lambda_{g}$	0
$\beta ,\phi ,p_{g},\Lambda_{r}$	0
$\beta ,\phi ,p_{g},\Lambda_{t}$	0
$\beta ,\phi ,p_{g},\Lambda_{g,r}$	0
$\beta ,\phi ,p_{g},\Lambda_{g,t}$	0
$\beta ,\phi ,p_{g},\Lambda_{r,t}$	0
$\beta ,\phi ,p_{g},\Lambda_{g,r,t}$	0
$\beta ,\phi ,p_{t},\Lambda$	0
$\beta ,\phi ,p_{t},\Lambda_{g}$	0
$\beta ,\phi ,p_{t},\Lambda_{r}$	0
$\beta ,\phi ,p_{t},\Lambda_{t}$	0
$\beta ,\phi ,p_{t},\Lambda_{g,r}$	0
$\beta ,\phi ,p_{t},\Lambda_{g,t}$	0
$\beta ,\phi ,p_{t},\Lambda_{r,t}$	0
$\beta ,\phi ,p_{t},\Lambda_{g,r,t}$	0
$\beta ,\phi ,p_{g,t},\Lambda$	0
$\beta ,\phi ,p_{g,t},\Lambda_{g}$	0
$\beta ,\phi ,p_{g,t},\Lambda_{r}$	0
$\beta ,\phi ,p_{g,t},\Lambda_{t}$	0
$\beta ,\phi ,p_{g,t},\Lambda_{g,r}$	0
$\beta ,\phi ,p_{g,t},\Lambda_{g,t}$	0
$\beta ,\phi ,p_{g,t},\Lambda_{r,t}$	0
$\beta ,\phi ,p_{g,t},\Lambda_{g,r,t}$	0
$\beta ,\phi_{g},p,\Lambda$	0
$\beta ,\phi_{g},p,\Lambda_{g}$	0
$\beta ,\phi_{g},p,\Lambda_{r}$	0
$\beta ,\phi_{g},p,\Lambda_{t}$	0
$\beta ,\phi_{g},p,\Lambda_{g,r}$	0
$\beta ,\phi_{g},p,\Lambda_{g,t}$	0
$\beta ,\phi_{g},p,\Lambda_{r,t}$	0
$\beta ,\phi_{g},p,\Lambda_{g,r,t}$	0
$\beta ,\phi_{g},p_{g},\Lambda$	0
$\beta ,\phi_{g},p_{g},\Lambda_{g}$	0
$\beta ,\phi_{g},p_{g},\Lambda_{r}$	0
$\beta ,\phi_{g},p_{g},\Lambda_{t}$	0
$\beta ,\phi_{g},p_{g},\Lambda_{g,r}$	0
$\beta ,\phi_{g},p_{g},\Lambda_{g,t}$	0
$\beta ,\phi_{g},p_{g},\Lambda_{r,t}$	0
$\beta ,\phi_{g},p_{g},\Lambda_{g,r,t}$	0
$\beta ,\phi_{g},p_{t},\Lambda$	0
$\beta ,\phi_{g},p_{t},\Lambda_{g}$	0
$\beta ,\phi_{g},p_{t},\Lambda_{r}$	0
$\beta ,\phi_{g},p_{t},\Lambda_{t}$	0
$\beta ,\phi_{g},p_{t},\Lambda_{g,t}$	0
$\beta ,\phi_{g},p_{t},\Lambda_{g,r}$	0
$\beta ,\phi_{g},p_{t},\Lambda_{r,t}$	0
$\beta ,\phi_{g},p_{t},\Lambda_{g,r,t}$	0
$\beta ,\phi_{g},p_{g,t},\Lambda$	0
$\beta ,\phi_{g},p_{g,t},\Lambda_{g}$	0
$\beta ,\phi_{g},p_{g,t},\Lambda_{r}$	0
$\beta ,\phi_{g},p_{g,t},\Lambda_{t}$	0
$\beta ,\phi_{g},p_{g,t},\Lambda_{g,r}$	0
$\beta ,\phi_{g},p_{g,t},\Lambda_{g,t}$	0
$\beta ,\phi_{g},p_{g,t},\Lambda_{r,t}$	0
$\beta ,\phi_{g},p_{g,t},\Lambda_{g,r,t}$	0
$\beta ,\phi_{t},p,\Lambda$	0
$\beta ,\phi_{t},p,\Lambda_{g}$	0
$\beta ,\phi_{t},p,\Lambda_{r}$	0
$\beta ,\phi_{t},p,\Lambda_{t}$	0
$\beta ,\phi_{t},p,\Lambda_{g,r}$	0
$\beta ,\phi_{t},p,\Lambda_{g,t}$	0
$\beta ,\phi_{t},p,\Lambda_{r,t}$	0
$\beta ,\phi_{t},p,\Lambda_{g,r,t}$	0
$\beta ,\phi_{t},p_{g},\Lambda$	0
$\beta ,\phi_{t},p_{g},\Lambda_{g}$	0
$\beta ,\phi_{t},p_{g},\Lambda_{r}$	0
$\beta ,\phi_{t},p_{g},\Lambda_{t}$	0
$\beta ,\phi_{t},p_{g},\Lambda_{g,r}$	0
$\beta ,\phi_{t},p_{g},\Lambda_{g,t}$	0
$\beta ,\phi_{t},p_{g},\Lambda_{r,t}$	0
$\beta ,\phi_{t},p_{g},\Lambda_{g,r,t}$	0
$\beta ,\phi_{t},p_{t},\Lambda$	0
$\beta ,\phi_{t},p_{t},\Lambda_{g}$	0
$\beta ,\phi_{t},p_{t},\Lambda_{r}$	0
$\beta ,\phi_{t},p_{t},\Lambda_{t}$	0
$\beta ,\phi_{t},p_{t},\Lambda_{g,r}$	0
$\beta ,\phi_{t},p_{t},\Lambda_{g,t}$	0
$\beta ,\phi_{t},p_{t},\Lambda_{r,t}$	0
$\beta ,\phi_{t},p_{t},\Lambda_{g,r,t}$	0
$\beta ,\phi_{t},p_{g,t},\Lambda$	0
$\beta ,\phi_{t},p_{g,t},\Lambda_{g}$	0
$\beta ,\phi_{t},p_{g,t},\Lambda_{r}$	0
$\beta ,\phi_{t},p_{g,t},\Lambda_{t}$	0
$\beta ,\phi_{t},p_{g,t},\Lambda_{g,r}$	0
$\beta ,\phi_{t},p_{g,t},\Lambda_{g,t}$	0
$\beta ,\phi_{t},p_{g,t},\Lambda_{r,t}$	0
$\beta ,\phi_{t},p_{g,t},\Lambda_{g,r,t}$	0
$\beta ,\phi_{g,t},p,\Lambda$	0
$\beta ,\phi_{g,t},p,\Lambda_{g}$	0
$\beta ,\phi_{g,t},p,\Lambda_{r}$	0
$\beta ,\phi_{g,t},p,\Lambda_{t}$	0
$\beta ,\phi_{g,t},p,\Lambda_{g,r}$	0
$\beta ,\phi_{g,t},p,\Lambda_{g,t}$	0
$\beta ,\phi_{g,t},p,\Lambda_{r,t}$	0
$\beta ,\phi_{g,t},p,\Lambda_{g,r,t}$	0
$\beta ,\phi_{g,t},p_{g},\Lambda$	0
$\beta ,\phi_{g,t},p_{g},\Lambda_{g}$	0
$\beta ,\phi_{g,t},p_{g},\Lambda_{r}$	0
$\beta ,\phi_{g,t},p_{g},\Lambda_{t}$	0
$\beta ,\phi_{g,t},p_{g},\Lambda_{g,r}$	0
$\beta ,\phi_{g,t},p_{g},\Lambda_{g,t}$	0
$\beta ,\phi_{g,t},p_{g},\Lambda_{r,t}$	0
$\beta ,\phi_{g,t},p_{g},\Lambda_{g,r,t}$	0
$\beta ,\phi_{g,t},p_{t},\Lambda$	0
$\beta ,\phi_{g,t},p_{t},\Lambda_{g}$	0
$\beta ,\phi_{g,t},p_{t},\Lambda_{r}$	0
$\beta ,\phi_{g,t},p_{t},\Lambda_{t}$	0
$\beta ,\phi_{g,t},p_{t},\Lambda_{g,r}$	0
$\beta ,\phi_{g,t},p_{t},\Lambda_{g,t}$	0
$\beta ,\phi_{g,t},p_{t},\Lambda_{r,t}$	0
$\beta ,\phi_{g,t},p_{t},\Lambda_{g,r,t}$	0
$\beta ,\phi_{g,t},p_{g,t},\Lambda$	0
$\beta ,\phi_{g,t},p_{g,t},\Lambda_{g}$	0
$\beta ,\phi_{g,t},p_{g,t},\Lambda_{r}$	0
$\beta ,\phi_{g,t},p_{g,t},\Lambda_{t}$	0
$\beta ,\phi_{g,t},p_{g,t},\Lambda_{g,r}$	0
$\beta ,\phi_{g,t},p_{g,t},\Lambda_{g,t}$	0
$\beta ,\phi_{g,t},p_{g,t},\Lambda_{r,t}$	0
$\beta ,\phi_{g,t},p_{g,t},\Lambda_{g,r,t}$	0
$\beta_{g},\phi ,p,\Lambda$	0
$\beta_{g},\phi ,p,\Lambda_{g}$	0
$\beta_{g},\phi ,p,\Lambda_{r}$	0
$\beta_{g},\phi ,p,\Lambda_{t}$	0
$\beta_{g},\phi ,p,\Lambda_{g,r}$	0
$\beta_{g},\phi ,p,\Lambda_{g,t}$	0
$\beta_{g},\phi ,p,\Lambda_{r,t}$	0
$\beta_{g},\phi ,p,\Lambda_{g,r,t}$	0
$\beta_{g},\phi ,p_{g},\Lambda$	0
$\beta_{g},\phi ,p_{g},\Lambda_{g}$	0
$\beta_{g},\phi ,p_{g},\Lambda_{r}$	0
$\beta_{g},\phi ,p_{g},\Lambda_{t}$	0
$\beta_{g},\phi ,p_{g},\Lambda_{g,r}$	0
$\beta_{g},\phi ,p_{g},\Lambda_{g,t}$	0
$\beta_{g},\phi ,p_{g},\Lambda_{r,t}$	0
$\beta_{g},\phi ,p_{g},\Lambda_{g,r,t}$	0
$\beta_{g},\phi ,p_{t},\Lambda$	0
$\beta_{g},\phi ,p_{t},\Lambda_{g}$	0
$\beta_{g},\phi ,p_{t},\Lambda_{r}$	0
$\beta_{g},\phi ,p_{t},\Lambda_{t}$	0
$\beta_{g},\phi ,p_{t},\Lambda_{g,r}$	0
$\beta_{g},\phi ,p_{t},\Lambda_{g,t}$	0
$\beta_{g},\phi ,p_{t},\Lambda_{r,t}$	0
$\beta_{g},\phi ,p_{t},\Lambda_{g,r,t}$	0
$\beta_{g},\phi ,p_{g,t},\Lambda$	0
$\beta_{g},\phi ,p_{g,t},\Lambda_{g}$	0
$\beta_{g},\phi ,p_{g,t},\Lambda_{r}$	0
$\beta_{g},\phi ,p_{g,t},\Lambda_{t}$	0
$\beta_{g},\phi ,p_{g,t},\Lambda_{g,r}$	0
$\beta_{g},\phi ,p_{g,t},\Lambda_{g,t}$	0
$\beta_{g},\phi ,p_{g,t},\Lambda_{r,t}$	0
$\beta_{g},\phi ,p_{g,t},\Lambda_{g,r,t}$	0
$\beta_{g},\phi_{g},p,\Lambda$	0
$\beta_{g},\phi_{g},p,\Lambda_{g}$	0
$\beta_{g},\phi_{g},p,\Lambda_{r}$	0
$\beta_{g},\phi_{g},p,\Lambda_{t}$	0
$\beta_{g},\phi_{g},p,\Lambda_{g,r}$	0
$\beta_{g},\phi_{g},p,\Lambda_{g,t}$	0
$\beta_{g},\phi_{g},p,\Lambda_{r,t}$	0
$\beta_{g},\phi_{g},p,\Lambda_{g,r,t}$	0
$\beta_{g},\phi_{g},p_{g},\Lambda$	0
$\beta_{g},\phi_{g},p_{g},\Lambda_{g}$	0
$\beta_{g},\phi_{g},p_{g},\Lambda_{r}$	0
$\beta_{g},\phi_{g},p_{g},\Lambda_{t}$	0
$\beta_{g},\phi_{g},p_{g},\Lambda_{g,r}$	0
$\beta_{g},\phi_{g},p_{g},\Lambda_{g,t}$	0
$\beta_{g},\phi_{g},p_{g},\Lambda_{r,t}$	0
$\beta_{g},\phi_{g},p_{g},\Lambda_{g,r,t}$	0
$\beta_{g},\phi_{g},p_{t},\Lambda$	0
$\beta_{g},\phi_{g},p_{t},\Lambda_{g}$	0
$\beta_{g},\phi_{g},p_{t},\Lambda_{r}$	0
$\beta_{g},\phi_{g},p_{t},\Lambda_{t}$	0
$\beta_{g},\phi_{g},p_{t},\Lambda_{g,r}$	0
$\beta_{g},\phi_{g},p_{t},\Lambda_{g,t}$	0
$\beta_{g},\phi_{g},p_{t},\Lambda_{r,t}$	0
$\beta_{g},\phi_{g},p_{t},\Lambda_{g,r,t}$	0
$\beta_{g},\phi_{g},p_{g,t},\Lambda$	0
$\beta_{g},\phi_{g},p_{g,t},\Lambda_{g}$	0
$\beta_{g},\phi_{g},p_{g,t},\Lambda_{r}$	0
$\beta_{g},\phi_{g},p_{g,t},\Lambda_{t}$	0
$\beta_{g},\phi_{g},p_{g,t},\Lambda_{g,r}$	0
$\beta_{g},\phi_{g},p_{g,t},\Lambda_{g,t}$	0
$\beta_{g},\phi_{g},p_{g,t},\Lambda_{r,t}$	0
$\beta_{g},\phi_{g},p_{g,t},\Lambda_{g,r,t}$	0
$\beta_{g},\phi_{t},p,\Lambda$	0
$\beta_{g},\phi_{t},p,\Lambda_{g}$	0
$\beta_{g},\phi_{t},p,\Lambda_{r}$	0
$\beta_{g},\phi_{t},p,\Lambda_{t}$	0
$\beta_{g},\phi_{t},p,\Lambda_{g,r}$	0
$\beta_{g},\phi_{t},p,\Lambda_{g,t}$	0
$\beta_{g},\phi_{t},p,\Lambda_{r,t}$	0
$\beta_{g},\phi_{t},p,\Lambda_{g,r,t}$	0
$\beta_{g},\phi_{t},p_{g},\Lambda$	0
$\beta_{g},\phi_{t},p_{g},\Lambda_{g}$	0
$\beta_{g},\phi_{t},p_{g},\Lambda_{r}$	0
$\beta_{g},\phi_{t},p_{g},\Lambda_{t}$	0
$\beta_{g},\phi_{t},p_{g},\Lambda_{g,r}$	0
$\beta_{g},\phi_{t},p_{g},\Lambda_{g,t}$	0
$\beta_{g},\phi_{t},p_{g},\Lambda_{r,t}$	0
$\beta_{g},\phi_{t},p_{g},\Lambda_{g,r,t}$	0
$\beta_{g},\phi_{t},p_{t},\Lambda$	0
$\beta_{g},\phi_{t},p_{t},\Lambda_{g}$	0
$\beta_{g},\phi_{t},p_{t},\Lambda_{r}$	0
$\beta_{g},\phi_{t},p_{t},\Lambda_{t}$	0
$\beta_{g},\phi_{t},p_{t},\Lambda_{g,r}$	0
$\beta_{g},\phi_{t},p_{t},\Lambda_{g,t}$	0
$\beta_{g},\phi_{t},p_{t},\Lambda_{r,t}$	0
$\beta_{g},\phi_{t},p_{t},\Lambda_{g,r,t}$	0
$\beta_{g},\phi_{t},p_{g,t},\Lambda$	0
$\beta_{g},\phi_{t},p_{g,t},\Lambda_{g}$	0
$\beta_{g},\phi_{t},p_{g,t},\Lambda_{r}$	0
$\beta_{g},\phi_{t},p_{g,t},\Lambda_{t}$	0
$\beta_{g},\phi_{t},p_{g,t},\Lambda_{g,r}$	0
$\beta_{g},\phi_{t},p_{g,t},\Lambda_{g,t}$	0
$\beta_{g},\phi_{t},p_{g,t},\Lambda_{r,t}$	0
$\beta_{g},\phi_{t},p_{g,t},\Lambda_{g,r,t}$	0
$\beta_{g},\phi_{g,t},p,\Lambda$	0
$\beta_{g},\phi_{g,t},p,\Lambda_{g}$	0
$\beta_{g},\phi_{g,t},p,\Lambda_{r}$	0
$\beta_{g},\phi_{g,t},p,\Lambda_{t}$	0
$\beta_{g},\phi_{g,t},p,\Lambda_{g,r}$	0
$\beta_{g},\phi_{g,t},p,\Lambda_{g,t}$	0
$\beta_{g},\phi_{g,t},p,\Lambda_{r,t}$	0
$\beta_{g},\phi_{g,t},p,\Lambda_{g,r,t}$	0
$\beta_{g},\phi_{g,t},p_{g},\Lambda$	0
$\beta_{g},\phi_{g,t},p_{g},\Lambda_{g}$	0
$\beta_{g},\phi_{g,t},p_{g},\Lambda_{r}$	0
$\beta_{g},\phi_{g,t},p_{g},\Lambda_{t}$	0
$\beta_{g},\phi_{g,t},p_{g},\Lambda_{g,r}$	0
$\beta_{g},\phi_{g,t},p_{g},\Lambda_{g,t}$	0
$\beta_{g},\phi_{g,t},p_{g},\Lambda_{r,t}$	0
$\beta_{g},\phi_{g,t},p_{g},\Lambda_{g,r,t}$	0
$\beta_{g},\phi_{g,t},p_{t},\Lambda$	0
$\beta_{g},\phi_{g,t},p_{t},\Lambda_{g}$	0
$\beta_{g},\phi_{g,t},p_{t},\Lambda_{r}$	0
$\beta_{g},\phi_{g,t},p_{t},\Lambda_{t}$	0
$\beta_{g},\phi_{g,t},p_{t},\Lambda_{g,r}$	0
$\beta_{g},\phi_{g,t},p_{t},\Lambda_{g,t}$	0
$\beta_{g},\phi_{g,t},p_{t},\Lambda_{r,t}$	0
$\beta_{g},\phi_{g,t},p_{t},\Lambda_{g,r,t}$	0
$\beta_{g},\phi_{g,t},p_{g,t},\Lambda$	0
$\beta_{g},\phi_{g,t},p_{g,t},\Lambda_{g}$	0
$\beta_{g},\phi_{g,t},p_{g,t},\Lambda_{r}$	0
$\beta_{g},\phi_{g,t},p_{g,t},\Lambda_{t}$	0
$\beta_{g},\phi_{g,t},p_{g,t},\Lambda_{g,r}$	0
$\beta_{g},\phi_{g,t},p_{g,t},\Lambda_{g,t}$	0
$\beta_{g},\phi_{g,t},p_{g,t},\Lambda_{r,t}$	0
$\beta_{g},\phi_{g,t},p_{g,t},\Lambda_{g,r,t}$	0
$\beta_{t},\phi ,p,\Lambda$	0
$\beta_{t},\phi ,p,\Lambda_{g}$	0
$\beta_{t},\phi ,p,\Lambda_{r}$	0
$\beta_{t},\phi ,p,\Lambda_{t}$	0
$\beta_{t},\phi ,p,\Lambda_{g,r}$	0
$\beta_{t},\phi ,p,\Lambda_{g,t}$	0
$\beta_{t},\phi ,p,\Lambda_{r,t}$	0
$\beta_{t},\phi ,p,\Lambda_{g,r,t}$	0
$\beta_{t},\phi ,p_{g},\Lambda$	0
$\beta_{t},\phi ,p_{g},\Lambda_{g}$	0
$\beta_{t},\phi ,p_{g},\Lambda_{r}$	0
$\beta_{t},\phi ,p_{g},\Lambda_{t}$	0
$\beta_{t},\phi ,p_{g},\Lambda_{g,r}$	0
$\beta_{t},\phi ,p_{g},\Lambda_{g,t}$	0
$\beta_{t},\phi ,p_{g},\Lambda_{r,t}$	0
$\beta_{t},\phi ,p_{g},\Lambda_{g,r,t}$	0
$\beta_{t},\phi ,p_{t},\Lambda$	0
$\beta_{t},\phi ,p_{t},\Lambda_{g}$	0
$\beta_{t},\phi ,p_{t},\Lambda_{r}$	0
$\beta_{t},\phi ,p_{t},\Lambda_{t}$	0
$\beta_{t},\phi ,p_{t},\Lambda_{g,r}$	0
$\beta_{t},\phi ,p_{t},\Lambda_{g,t}$	0
$\beta_{t},\phi ,p_{t},\Lambda_{r,t}$	0
$\beta_{t},\phi ,p_{t},\Lambda_{g,r,t}$	0
$\beta_{t},\phi ,p_{g,t},\Lambda$	0
$\beta_{t},\phi ,p_{g,t},\Lambda_{g}$	0
$\beta_{t},\phi ,p_{g,t},\Lambda_{r}$	0
$\beta_{t},\phi ,p_{g,t},\Lambda_{t}$	0
$\beta_{t},\phi ,p_{g,t},\Lambda_{g,r}$	0
$\beta_{t},\phi ,p_{g,t},\Lambda_{g,t}$	0
$\beta_{t},\phi ,p_{g,t},\Lambda_{r,t}$	0
$\beta_{t},\phi ,p_{g,t},\Lambda_{g,r,t}$	0
$\beta_{t},\phi_{g},p,\Lambda$	0
$\beta_{t},\phi_{g},p,\Lambda_{g}$	0
$\beta_{t},\phi_{g},p,\Lambda_{r}$	0
$\beta_{t},\phi_{g},p,\Lambda_{t}$	0
$\beta_{t},\phi_{g},p,\Lambda_{g,t}$	0
$\beta_{t},\phi_{g},p,\Lambda_{g,r}$	0
$\beta_{t},\phi_{g},p,\Lambda_{r,t}$	0
$\beta_{t},\phi_{g},p,\Lambda_{g,r,t}$	0
$\beta_{t},\phi_{g},p_{t},\Lambda_{g}$	0
$\beta_{t},\phi_{g},p_{t},\Lambda_{r}$	0
$\beta_{t},\phi_{g},p_{t},\Lambda_{t}$	0
$\beta_{t},\phi_{g},p_{t},\Lambda_{g,r}$	0
$\beta_{t},\phi_{g},p_{t},\Lambda_{g,t}$	0
$\beta_{t},\phi_{g},p_{t},\Lambda_{r,t}$	0
$\beta_{t},\phi_{g},p_{t},\Lambda_{g,r,t}$	0
$\beta_{t},\phi_{g},p_{g},\Lambda$	0
$\beta_{t},\phi_{g},p_{g},\Lambda_{g}$	0
$\beta_{t},\phi_{g},p_{g},\Lambda_{r}$	0
$\beta_{t},\phi_{g},p_{g},\Lambda_{t}$	0
$\beta_{t},\phi_{g},p_{g},\Lambda_{g,r}$	0
$\beta_{t},\phi_{g},p_{g},\Lambda_{g,t}$	0
$\beta_{t},\phi_{g},p_{g},\Lambda_{r,t}$	0
$\beta_{t},\phi_{g},p_{g},\Lambda_{g,r,t}$	0
$\beta_{t},\phi_{g},p_{g,t},\Lambda$	0
$\beta_{t},\phi_{g},p_{g,t},\Lambda_{g}$	0
$\beta_{t},\phi_{g},p_{g,t},\Lambda_{r}$	0
$\beta_{t},\phi_{g},p_{g,t},\Lambda_{t}$	0
$\beta_{t},\phi_{g},p_{g,t},\Lambda_{g,r}$	0
$\beta_{t},\phi_{g},p_{g,t},\Lambda_{g,t}$	0
$\beta_{t},\phi_{g},p_{g,t},\Lambda_{r,t}$	0
$\beta_{t},\phi_{g},p_{g,t},\Lambda_{g,r,t}$	0
$\beta_{t},\phi_{t},p,\Lambda$	0
$\beta_{t},\phi_{t},p,\Lambda_{g}$	0
$\beta_{t},\phi_{t},p,\Lambda_{r}$	0
$\beta_{t},\phi_{t},p,\Lambda_{t}$	0
$\beta_{t},\phi_{t},p,\Lambda_{g,r}$	0
$\beta_{t},\phi_{t},p,\Lambda_{g,t}$	0
$\beta_{t},\phi_{t},p,\Lambda_{r,t}$	0
$\beta_{t},\phi_{t},p,\Lambda_{g,r,t}$	0
$\beta_{t},\phi_{t},p_{g},\Lambda$	0
$\beta_{t},\phi_{t},p_{g},\Lambda_{g}$	0
$\beta_{t},\phi_{t},p_{g},\Lambda_{r}$	0
$\beta_{t},\phi_{t},p_{g},\Lambda_{t}$	0
$\beta_{t},\phi_{t},p_{g},\Lambda_{g,t}$	0
$\beta_{t},\phi_{t},p_{g},\Lambda_{r,t}$	0
$\beta_{t},\phi_{t},p_{g},\Lambda_{g,r,t}$	0
$\beta_{t},\phi_{t},p_{t},\Lambda$	1	N, Λ, $\phi_{1},\cdots ,\phi_{T‐2}$, $\beta_{2},\cdots ,\beta_{T‐2}$, $p_{2},\cdots ,p_{T‐1}$,
		$\beta_{0}p_{1}$, $\beta_{0}\phi_{1}+\beta_{1}$, $\phi_{T‐1}p_{T}$, $p_{T}\beta_{T‐1}$
$\beta_{t},\phi_{t},p_{t},\Lambda_{g}$	1	$N_{g}$, $\Lambda_{g}$, $\phi_{1},\cdots ,\phi_{T‐2}$, $\beta_{2},\cdots ,\beta_{T‐2}$, $p_{2},\cdots ,p_{T‐1}$,
		$\beta_{0}p_{1}$, $\beta_{0}\phi_{1}+\beta_{1}$, $\phi_{T‐1}p_{T}$, $p_{T}\beta_{T‐1}$
$\beta_{t},\phi_{t},p_{t},\Lambda_{r}$	1	N, Λ, $\phi_{1},\cdots ,\phi_{T‐2}$, $\beta_{2},\cdots ,\beta_{T‐2}$, $p_{2},\cdots ,p_{T‐1}$,
		$\beta_{0}p_{1}$, $\beta_{0}\phi_{1}+\beta_{1}$, $\phi_{T‐1}p_{T}$, $p_{T}\beta_{T‐1}$
$\beta_{t},\phi_{t},p_{t},\Lambda_{t}$	1	N, $\Lambda_{t}$, $\phi_{1},\cdots ,\phi_{T‐2}$, $\beta_{2},\cdots ,\beta_{T‐2}$, $p_{2},\cdots ,p_{T‐1}$,
		$\beta_{0}p_{1}$, $\beta_{0}\phi_{1}+\beta_{1}$, $\phi_{T‐1}p_{T}$, $p_{T}\beta_{T‐1}$
$\beta_{t},\phi_{t},p_{t},\Lambda_{g,r}$	1	$N_{g}$, $\Lambda_{g,r}$, $\phi_{1},\cdots ,\phi_{T‐2}$, $\beta_{2},\cdots ,\beta_{T‐2}$, $p_{2},\cdots ,p_{T‐1}$,
		$\beta_{0}p_{1}$, $\beta_{0}\phi_{1}+\beta_{1}$, $\phi_{T‐1}p_{T}$, $p_{T}\beta_{T‐1}$
$\beta_{t},\phi_{t},p_{t},\Lambda_{g,t}$	1	$N_{g}$, Λ, $\phi_{1},\cdots ,\phi_{T‐2}$, $\beta_{2},\cdots ,\beta_{T‐2}$, $p_{2},\cdots ,p_{T‐1}$,
		$\beta_{0}p_{1}$, $\beta_{0}\phi_{1}+\beta_{1}$, $\phi_{T‐1}p_{T}$, $p_{T}\beta_{T‐1}$
$\beta_{t},\phi_{t},p_{t},\Lambda_{r,t}$	1	N, Λr,t, $\phi_{1},\cdots ,\phi_{T‐2}$, $\beta_{2},\cdots ,\beta_{T‐2}$, $p_{2},\cdots ,p_{T‐1}$,
		$\beta_{0}p_{1}$, $\beta_{0}\phi_{1}+\beta_{1}$, $\phi_{T‐1}p_{T}$, $p_{T}\beta_{T‐1}$
$\beta_{t},\phi_{t},p_{t},\Lambda_{g,r,t}$	1	$N_{g}$, $\Lambda_{g,r,t}$, $\phi_{1},\cdots ,\phi_{T‐2}$, $\beta_{2},\cdots ,\beta_{T‐2}$, $p_{2},\cdots ,p_{T‐1}$,
		$\beta_{0}p_{1}$, $\beta_{0}\phi_{1}+\beta_{1}$, $\phi_{T‐1}p_{T}$, $p_{T}\beta_{T‐1}$
$\beta_{t},\phi_{t},p_{g,t},\Lambda$	1	$N_{g}$, Λ, $\phi_{1},\cdots ,\phi_{T‐2}$, $\beta_{2},\cdots ,\beta_{T‐2}$, $p_{g,2},\cdots ,p_{g,T‐1}$,
		$\beta_{0}p_{g,1}$, $\beta_{0}\phi_{1}+\beta_{1}$, $\phi_{T‐1}p_{g,T}$, $\beta_{T‐1}/\phi_{T‐1}$
$\beta_{t},\phi_{t},p_{g,t},\Lambda_{g}$	1	$N_{g}$, $\Lambda_{g}$, $\phi_{1},\cdots ,\phi_{T‐2}$, $\beta_{2},\cdots ,\beta_{T‐2}$, $p_{g,2},\cdots ,p_{g,T‐1}$,
		$\beta_{0}p_{g,1}$, $\beta_{0}\phi_{1}+\beta_{1}$, $\phi_{T‐1}p_{g,T}$, $\beta_{T‐1}/\phi_{T‐1}$
$\beta_{t},\phi_{t},p_{g,t},\Lambda_{r}$	1	$N_{g}$, $\Lambda_{r}$, $\phi_{1},\cdots ,\phi_{T‐2}$, $\beta_{2},\cdots ,\beta_{T‐2}$, $p_{g,2},\cdots ,p_{g,T‐1}$,
		$\beta_{0}p_{g,1}$, $\beta_{0}\phi_{1}+\beta_{1}$, $\phi_{T‐1}p_{g,T}$, $\beta_{T‐1}/\phi_{T‐1}$
$\beta_{t},\phi_{t},p_{g,t},\Lambda_{t}$	1	$N_{g}$, $\Lambda_{t}$, $\phi_{1},\cdots ,\phi_{T‐2}$, $\beta_{2},\cdots ,\beta_{T‐2}$, $p_{g,2},\cdots ,p_{g,T‐1}$,
		$\beta_{0}p_{g,1}$, $\beta_{0}\phi_{1}+\beta_{1}$, $\phi_{T‐1}p_{g,T}$,$\beta_{T‐1}/\phi_{T‐1}$
$\beta_{t},\phi_{t},p_{g,t},\Lambda_{g,r}$	1	$N_{g}$, $\Lambda_{g,r}$, $\phi_{1},\cdots ,\phi_{T‐2}$, $\beta_{2},\cdots ,\beta_{T‐2}$, $p_{g,2},\cdots ,p_{g,T‐1}$,
		$\beta_{0}p_{g,1}$, $\beta_{0}\phi_{1}+\beta_{1}$, $\phi_{T‐1}p_{g,T}$,$\beta_{T‐1}/\phi_{T‐1}$
$\beta_{t},\phi_{t},p_{g,t},\Lambda_{g,t}$	1	$N_{g}$, $\Lambda_{g,t}$, $\phi_{1},\cdots ,\phi_{T‐2}$, $\beta_{2},\cdots ,\beta_{T‐2}$, $p_{g,2},\cdots ,p_{g,T‐1}$,
		$\beta_{0}p_{g,1}$, $\beta_{0}\phi_{1}+\beta_{1}$, $\phi_{T‐1}p_{g,T}$,$\beta_{T‐1}/\phi_{T‐1}$
$\beta_{t},\phi_{t},p_{g,t},\Lambda_{r,t}$	1	$N_{g}$, $\Lambda_{r,t}$, $\phi_{1},\cdots ,\phi_{T‐2}$, $\beta_{2},\cdots ,\beta_{T‐2}$, $p_{g,2},\cdots ,p_{g,T‐1}$,
		$\beta_{0}p_{g,1}$, $\beta_{0}\phi_{1}+\beta_{1}$, $\phi_{T‐1}p_{g,T}$,$\beta_{T‐1}/\phi_{T‐1}$
$\beta_{t},\phi_{t},p_{g,t},\Lambda_{g,r,t}$	1	$N_{g}$, $\Lambda_{g,r,t}$, $\phi_{1},\cdots ,\phi_{T‐2}$, $\beta_{2},\cdots ,\beta_{T‐2}$, $p_{g,2},\cdots ,p_{g,T‐1}$,
		$\beta_{0}p_{g,1}$, $\beta_{0}\phi_{1}+\beta_{1}$, $\phi_{T‐1}p_{g,T}$,$\beta_{T‐1}/\phi_{T‐1}$
$\beta_{t},\phi_{g,t},p,\Lambda$	0
$\beta_{t},\phi_{g,t},p,\Lambda_{g}$	0
$\beta_{t},\phi_{g,t},p,\Lambda_{r}$	0
$\beta_{t},\phi_{g,t},p,\Lambda_{t}$	0
$\beta_{t},\phi_{g,t},p,\Lambda_{g,r}$	0
$\beta_{t},\phi_{g,t},p,\Lambda_{g,t}$	0
$\beta_{t},\phi_{g,t},p,\Lambda_{r,t}$	0
$\beta_{t},\phi_{g,t},p,\Lambda_{g,r,t}$	0
$\beta_{t},\phi_{g,t},p_{g},\Lambda$	0
$\beta_{t},\phi_{g,t},p_{g},\Lambda_{g}$	0
$\beta_{t},\phi_{g,t},p_{g},\Lambda_{r}$	0
$\beta_{t},\phi_{g,t},p_{g},\Lambda_{t}$	0
$\beta_{t},\phi_{g,t},p_{g},\Lambda_{g,r}$	0
$\beta_{t},\phi_{g,t},p_{g},\Lambda_{g,t}$	0
$\beta_{t},\phi_{g,t},p_{g},\Lambda_{r,t}$	0
$\beta_{t},\phi_{g,t},p_{g},\Lambda_{g,r,t}$	0
$\beta_{t},\phi_{g,t},p_{t},\Lambda$	0
$\beta_{t},\phi_{g,t},p_{t},\Lambda_{g}$	0
$\beta_{t},\phi_{g,t},p_{t},\Lambda_{r}$	0
$\beta_{t},\phi_{g,t},p_{t},\Lambda_{t}$	0
$\beta_{t},\phi_{g,t},p_{t},\Lambda_{g,r}$	0
$\beta_{t},\phi_{g,t},p_{t},\Lambda_{g,t}$	0
$\beta_{t},\phi_{g,t},p_{t},\Lambda_{r,t}$	0
$\beta_{t},\phi_{g,t},p_{t},\Lambda_{g,r,t}$	0
$\beta_{t},\phi_{g,t},p_{g,t},\Lambda$	0
$\beta_{t},\phi_{g,t},p_{g,t},\Lambda_{g}$	0
$\beta_{t},\phi_{g,t},p_{g,t},\Lambda_{r}$	0
$\beta_{t},\phi_{g,t},p_{g,t},\Lambda_{t}$	0
$\beta_{t},\phi_{g,t},p_{g,t},\Lambda_{g,r}$	0
$\beta_{t},\phi_{g,t},p_{g,t},\Lambda_{g,t}$	0
$\beta_{t},\phi_{g,t},p_{g,t},\Lambda_{r,t}$	0
$\beta_{t},\phi_{g,t},p_{g,t},\Lambda_{g,r,t}$	0
$\beta_{g,t},\phi ,p,\Lambda$	0
$\beta_{g,t},\phi ,p,\Lambda_{g}$	0
$\beta_{g,t},\phi ,p,\Lambda_{r}$	0
$\beta_{g,t},\phi ,p,\Lambda_{t}$	0
$\beta_{g,t},\phi ,p,\Lambda_{g,r}$	0
$\beta_{g,t},\phi ,p,\Lambda_{g,t}$	0
$\beta_{g,t},\phi ,p,\Lambda_{r,t}$	0
$\beta_{g,t},\phi ,p,\Lambda_{g,r,t}$	0
$\beta_{g,t},\phi ,p_{g},\Lambda$	0
$\beta_{g,t},\phi ,p_{g},\Lambda_{g}$	0
$\beta_{g,t},\phi ,p_{g},\Lambda_{r}$	0
$\beta_{g,t},\phi ,p_{g},\Lambda_{t}$	0
$\beta_{g,t},\phi ,p_{g},\Lambda_{g,r}$	0
$\beta_{g,t},\phi ,p_{g},\Lambda_{g,t}$	0
$\beta_{g,t},\phi ,p_{g},\Lambda_{r,t}$	0
$\beta_{g,t},\phi ,p_{g},\Lambda_{g,r,t}$	0
$\beta_{g,t},\phi ,p_{t},\Lambda$	0
$\beta_{g,t},\phi ,p_{t},\Lambda_{g}$	0
$\beta_{g,t},\phi ,p_{t},\Lambda_{r}$	0
$\beta_{g,t},\phi ,p_{t},\Lambda_{t}$	0
$\beta_{g,t},\phi ,p_{t},\Lambda_{g,r}$	0
$\beta_{g,t},\phi ,p_{t},\Lambda_{g,t}$	0
$\beta_{g,t},\phi ,p_{t},\Lambda_{r,t}$	0
$\beta_{g,t},\phi ,p_{t},\Lambda_{g,r,t}$	0
$\beta_{g,t},\phi ,p_{g,t},\Lambda$	0
$\beta_{g,t},\phi ,p_{g,t},\Lambda_{g}$	0
$\beta_{g,t},\phi ,p_{g,t},\Lambda_{r}$	0
$\beta_{g,t},\phi ,p_{g,t},\Lambda_{t}$	0
$\beta_{g,t},\phi ,p_{g,t},\Lambda_{g,r}$	0
$\beta_{g,t},\phi ,p_{g,t},\Lambda_{g,t}$	0
$\beta_{g,t},\phi ,p_{g,t},\Lambda_{r,t}$	0
$\beta_{g,t},\phi ,p_{g,t},\Lambda_{g,r,t}$	0
$\beta_{g,t},\phi_{g},p,\Lambda$	0
$\beta_{g,t},\phi_{g},p,\Lambda_{g}$	0
$\beta_{g,t},\phi_{g},p,\Lambda_{r}$	0
$\beta_{g,t},\phi_{g},p,\Lambda_{t}$	0
$\beta_{g,t},\phi_{g},p,\Lambda_{g,r}$	0
$\beta_{g,t},\phi_{g},p,\Lambda_{g,t}$	0
$\beta_{g,t},\phi_{g},p,\Lambda_{r,t}$	0
$\beta_{g,t},\phi_{g},p,\Lambda_{g,r,t}$	0
$\beta_{g,t},\phi_{g},p_{g},\Lambda$	0
$\beta_{g,t},\phi_{g},p_{g},\Lambda_{g}$	0
$\beta_{g,t},\phi_{g},p_{g},\Lambda_{r}$	0
$\beta_{g,t},\phi_{g},p_{g},\Lambda_{t}$	0
$\beta_{g,t},\phi_{g},p_{g},\Lambda_{g,r}$	0
$\beta_{g,t},\phi_{g},p_{g},\Lambda_{g,t}$	0
$\beta_{g,t},\phi_{g},p_{g},\Lambda_{r,t}$	0
$\beta_{g,t},\phi_{g},p_{g},\Lambda_{g,r,t}$	0
$\beta_{g,t},\phi_{g},p_{t},\Lambda$	0
$\beta_{g,t},\phi_{g},p_{t},\Lambda_{g}$	0
$\beta_{g,t},\phi_{g},p_{t},\Lambda_{r}$	0
$\beta_{g,t},\phi_{g},p_{t},\Lambda_{t}$	0
$\beta_{g,t},\phi_{g},p_{t},\Lambda_{g,r}$	0
$\beta_{g,t},\phi_{g},p_{t},\Lambda_{g,t}$	0
$\beta_{g,t},\phi_{g},p_{t},\Lambda_{r,t}$	0
$\beta_{g,t},\phi_{g},p_{t},\Lambda_{g,r,t}$	0
$\beta_{g,t},\phi_{g},p_{g,t},\Lambda$	0
$\beta_{g,t},\phi_{g},p_{g,t},\Lambda_{g}$	0
$\beta_{g,t},\phi_{g},p_{g,t},\Lambda_{r}$	0
$\beta_{g,t},\phi_{g},p_{g,t},\Lambda_{t}$	0
$\beta_{g,t},\phi_{g},p_{g,t},\Lambda_{g,r}$	0
$\beta_{g,t},\phi_{g},p_{g,t},\Lambda_{g,t}$	0
$\beta_{g,t},\phi_{g},p_{g,t},\Lambda_{r,t}$	0
$\beta_{g,t},\phi_{g},p_{g,t},\Lambda_{g,r,t}$	0
$\beta_{g,t},\phi_{t},p,\Lambda$	0
$\beta_{g,t},\phi_{t},p,\Lambda_{g}$	0
$\beta_{g,t},\phi_{t},p,\Lambda_{r}$	0
$\beta_{g,t},\phi_{t},p,\Lambda_{t}$	0
$\beta_{g,t},\phi_{t},p,\Lambda_{g,r}$	0
$\beta_{g,t},\phi_{t},p,\Lambda_{g,t}$	0
$\beta_{g,t},\phi_{t},p,\Lambda_{r,t}$	0
$\beta_{g,t},\phi_{t},p,\Lambda_{g,r,t}$	0
$\beta_{g,t},\phi_{t},p_{g},\Lambda$	0
$\beta_{g,t},\phi_{t},p_{g},\Lambda_{g}$	0
$\beta_{g,t},\phi_{t},p_{g},\Lambda_{r}$	0
$\beta_{g,t},\phi_{t},p_{g},\Lambda_{t}$	0
$\beta_{g,t},\phi_{t},p_{g},\Lambda_{g,r}$	0
$\beta_{g,t},\phi_{t},p_{g},\Lambda_{g,t}$	0
$\beta_{g,t},\phi_{t},p_{g},\Lambda_{r,t}$	0
$\beta_{g,t},\phi_{t},p_{g},\Lambda_{g,r,t}$	0
$\beta_{g,t},\phi_{t},p_{t},\Lambda$	0
$\beta_{g,t},\phi_{t},p_{t},\Lambda_{g}$	0
$\beta_{g,t},\phi_{t},p_{t},\Lambda_{r}$	0
$\beta_{g,t},\phi_{t},p_{t},\Lambda_{t}$	0
$\beta_{g,t},\phi_{t},p_{t},\Lambda_{g,r}$	0
$\beta_{g,t},\phi_{t},p_{t},\Lambda_{g,t}$	0
$\beta_{g,t},\phi_{t},p_{t},\Lambda_{r,t}$	0
$\beta_{g,t},\phi_{t},p_{t},\Lambda_{g,r,t}$	0
$\beta_{g,t},\phi_{t},p_{g,t},\Lambda$	1	$N_{g}$, Λ, $\phi_{1},\cdots ,\phi_{T‐2}$, $\beta_{g,2},\cdots ,\beta_{g,T‐2}$, $p_{g,2},\cdots ,p_{g,T‐1}$,
		$\beta_{g,0}p_{g,1}$,$\beta_{g,0}\phi_{1}+\beta_{g,1}$, $p_{g,T}\beta_{g,T‐1}$,
		$\phi_{1,T‐1}p_{1,T},\cdots ,\phi_{G‐1,T‐1}p_{G‐1,T}$, $f(\beta_{g,i},\phi_{1})$
$\beta_{g,t},\phi_{t},p_{g,t},\Lambda_{g}$	1	$N_{g}$, $\Lambda_{g}$, $\phi_{1},\cdots ,\phi_{T‐2}$, $\beta_{g,2},\cdots ,\beta_{g,T‐2}$, $p_{g,2},\cdots ,p_{g,T‐1}$,
		$\beta_{g,0}p_{g,1}$,$\beta_{g,0}\phi_{1}+\beta_{g,1}$, $p_{g,T}\beta_{g,T‐1}$,
		$\phi_{1,T‐1}p_{1,T},\cdots ,\phi_{G‐1,T‐1}p_{G‐1,T}$, $f(\beta_{g,i},\phi_{1})$
$\beta_{g,t},\phi_{t},p_{g,t},\Lambda_{r}$	1	$N_{g}$, $\Lambda_{r}$, $\phi_{1},\cdots ,\phi_{T‐2}$, $\beta_{g,2},\cdots ,\beta_{g,T‐2}$, $p_{g,2},\cdots ,p_{g,T‐1}$,
		$\beta_{g,0}p_{g,1}$,$\beta_{g,0}\phi_{1}+\beta_{g,1}$, $p_{g,T}\beta_{g,T‐1}$,
		$\phi_{1,T‐1}p_{1,T},\cdots ,\phi_{G‐1,T‐1}p_{G‐1,T}$, $f(\beta_{g,i},\phi_{1})$
$\beta_{g,t},\phi_{t},p_{g,t},\Lambda_{t}$	1	$N_{g}$, $\Lambda_{t}$, $\phi_{1},\cdots ,\phi_{T‐2}$, $\beta_{g,2},\cdots ,\beta_{g,T‐2}$, $p_{g,2},\cdots ,p_{g,T‐1}$,
		$\beta_{g,0}p_{g,1}$,$\beta_{g,0}\phi_{1}+\beta_{g,1}$, $p_{g,T}\beta_{g,T‐1}$,
		$\phi_{1,T‐1}p_{1,T},\cdots ,\phi_{G‐1,T‐1}p_{G‐1,T}$, $f(\beta_{g,i},\phi_{1})$
$\beta_{g,t},\phi_{t},p_{g,t},\Lambda_{g,t}$	1	$N_{g}$, $\Lambda_{g,t}$, $\phi_{1},\cdots ,\phi_{T‐2}$, $\beta_{g,2},\cdots ,\beta_{g,T‐2}$, $p_{g,2},\cdots ,p_{g,T‐1}$,
		$\beta_{g,0}p_{g,1}$,$\beta_{g,0}\phi_{1}+\beta_{g,1}$, $p_{g,T}\beta_{g,T‐1}$,
		$\phi_{1,T‐1}p_{1,T},\cdots ,\phi_{G‐1,T‐1}p_{G‐1,T}$, $f(\beta_{g,i},\phi_{1})$
$\beta_{g,t},\phi_{t},p_{g,t},\Lambda_{g,r}$	1	$N_{g}$, $\Lambda_{g,r}$, $\phi_{1},\cdots ,\phi_{T‐2}$, $\beta_{g,2},\cdots ,\beta_{g,T‐2}$, $p_{g,2},\cdots ,p_{g,T‐1}$,
		$\beta_{g,0}p_{g,1}$,$\beta_{g,0}\phi_{1}+\beta_{g,1}$, $p_{g,T}\beta_{g,T‐1}$,
		$\phi_{1,T‐1}p_{1,T},\cdots ,\phi_{G‐1,T‐1}p_{G‐1,T}$, $f(\beta_{g,i},\phi_{1})$
$\beta_{g,t},\phi_{t},p_{g,t},\Lambda_{r,t}$	1	$N_{g}$, $\Lambda_{r,t}$, $\phi_{1},\cdots ,\phi_{T‐2}$, $\beta_{g,2},\cdots ,\beta_{g,T‐2}$, $p_{g,2},\cdots ,p_{g,T‐1}$,
		$\beta_{g,0}p_{g,1}$,$\beta_{g,0}\phi_{1}+\beta_{g,1}$, $p_{g,T}\beta_{g,T‐1}$,
		$\phi_{1,T‐1}p_{1,T},\cdots ,\phi_{G‐1,T‐1}p_{G‐1,T}$, $f(\beta_{g,i},\phi_{1})$
$\beta_{g,t},\phi_{t},p_{g,t},\Lambda_{g,r,t}$	1	$N_{g}$, $\Lambda_{g,r,t}$, $\phi_{1},\cdots ,\phi_{T‐2}$, $\beta_{g,2},\cdots ,\beta_{g,T‐2}$, $p_{g,2},\cdots ,p_{g,T‐1}$,
		$\beta_{g,0}p_{g,1}$,$\beta_{g,0}\phi_{1}+\beta_{g,1}$, $p_{g,T}\beta_{g,T‐1}$,
		$\phi_{1,T‐1}p_{1,T},\cdots ,\phi_{G‐1,T‐1}p_{G‐1,T}$,$f(\beta_{g,i},\phi_{1})$
$\beta_{g,t},\phi_{g,t},p,\Lambda$	0
$\beta_{g,t},\phi_{g,t},p,\Lambda_{g}$	0
$\beta_{g,t},\phi_{g,t},p,\Lambda_{r}$	0
$\beta_{g,t},\phi_{g,t},p,\Lambda_{t}$	0
$\beta_{g,t},\phi_{g,t},p,\Lambda_{g,r}$	0
$\beta_{g,t},\phi_{g,t},p,\Lambda_{g,t}$	0
$\beta_{g,t},\phi_{g,t},p,\Lambda_{r,t}$	0
$\beta_{g,t},\phi_{g,t},p,\Lambda_{g,r,t}$	0
$\beta_{g,t},\phi_{g,t},p_{g},\Lambda$	0
$\beta_{g,t},\phi_{g,t},p_{g},\Lambda_{g}$	0
$\beta_{g,t},\phi_{g,t},p_{g},\Lambda_{r}$	0
$\beta_{g,t},\phi_{g,t},p_{g},\Lambda_{t}$	0
$\beta_{g,t},\phi_{g,t},p_{g},\Lambda_{g,r}$	0
$\beta_{g,t},\phi_{g,t},p_{g},\Lambda_{g,t}$	0
$\beta_{g,t},\phi_{g,t},p_{g},\Lambda_{r,t}$	0
$\beta_{g,t},\phi_{g,t},p_{g},\Lambda_{g,r,t}$	0
$\beta_{g,t},\phi_{g,t},p_{t},\Lambda$	0
$\beta_{g,t},\phi_{g,t},p_{t},\Lambda_{g}$	0
$\beta_{g,t},\phi_{g,t},p_{t},\Lambda_{r}$	0
$\beta_{g,t},\phi_{g,t},p_{t},\Lambda_{t}$	0
$\beta_{g,t},\phi_{g,t},p_{t},\Lambda_{g,r}$	0
$\beta_{g,t},\phi_{g,t},p_{t},\Lambda_{g,t}$	0
$\beta_{g,t},\phi_{g,t},p_{t},\Lambda_{r,t}$	0
$\beta_{g,t},\phi_{g,t},p_{t},\Lambda_{g,r,t}$	0
$\beta_{g,t},\phi_{g,t},p_{g,t},\Lambda$	G	$N_{g}$, Λ, $\phi_{g,1},\cdots ,\phi_{g,T‐2}$,$\beta_{g,2},\cdots ,\beta_{g,T‐2}$, $p_{g,2},\cdots ,p_{g,T‐1}$,
		$\beta_{g,0}p_{g,1}$, $\beta_{g,0}\phi_{g,1}+\beta_{g,1}$, $\phi_{g,T‐1}p_{g,T}$,$p_{g,T}\beta_{g,T‐1}$
$\beta_{g,t},\phi_{g,t},p_{g,t},\Lambda_{g}$	G	$N_{g}$, $\Lambda_{g}$, $\phi_{g,1},\cdots ,\phi_{g,T‐2}$,$\beta_{g,2},\cdots ,\beta_{g,T‐2}$, $p_{g,2},\cdots ,p_{g,T‐1}$,
		$\beta_{g,0}p_{g,1}$, $\beta_{g,0}\phi_{g,1}+\beta_{g,1}$, $\phi_{g,T‐1}p_{g,T}$,$p_{g,T}\beta_{g,T‐1}$
$\beta_{g,t},\phi_{g,t},p_{g,t},\Lambda_{r}$	G	$N_{g}$, $\Lambda_{r}$, $\phi_{g,1},\cdots ,\phi_{g,T‐2}$,$\beta_{g,2},\cdots ,\beta_{g,T‐2}$, $p_{g,2},\cdots ,p_{g,T‐1}$,
		$\beta_{g,0}p_{g,1}$, $\beta_{g,0}\phi_{g,1}+\beta_{g,1}$, $\phi_{g,T‐1}p_{g,T}$,$p_{g,T}\beta_{g,T‐1}$
$\beta_{g,t},\phi_{g,t},p_{g,t},\Lambda_{t}$	G	$N_{g}$, $\Lambda_{t}$, $\phi_{g,1},\cdots ,\phi_{g,T‐2}$,$\beta_{g,2},\cdots ,\beta_{g,T‐2}$, $p_{g,2},\cdots ,p_{g,T‐1}$,
		$\beta_{g,0}p_{g,1}$, $\beta_{g,0}\phi_{g,1}+\beta_{g,1}$, $\phi_{g,T‐1}p_{g,T}$, $p_{g,T}\beta_{g,T‐1}$
$\beta_{g,t},\phi_{g,t},p_{g,t},\Lambda_{g,r}$	G	$N_{g}$, $\Lambda_{g,r}$, $\phi_{g,1},\cdots ,\phi_{g,T‐2}$,$\beta_{g,2},\cdots ,\beta_{g,T‐2}$, $p_{g,2},\cdots ,p_{g,T‐1}$,
		$\beta_{g,0}p_{g,1}$, $\beta_{g,0}\phi_{g,1}+\beta_{g,1}$, $\phi_{g,T‐1}p_{g,T}$,$p_{g,T}\beta_{g,T‐1}$
$\beta_{g,t},\phi_{g,t},p_{g,t},\Lambda_{g,t}$	G	$N_{g}$, $\Lambda_{g,t}$, $\phi_{g,1},\cdots ,\phi_{g,T‐2}$,$\beta_{g,2},\cdots ,\beta_{g,T‐2}$, $p_{g,2},\cdots ,p_{g,T‐1}$,
		$\beta_{g,0}p_{g,1}$, $\beta_{g,0}\phi_{g,1}+\beta_{g,1}$, $\phi_{g,T‐1}p_{g,T}$,$p_{g,T}\beta_{g,T‐1}$
$\beta_{g,t},\phi_{g,t},p_{g,t},\Lambda_{r,t}$	G	$N_{g}$, $\Lambda_{r,t}$, $\phi_{g,1},\cdots ,\phi_{g,T‐2}$,$\beta_{g,2},\cdots ,\beta_{g,T‐2}$, $p_{g,2},\cdots ,p_{g,T‐1}$,
		$\beta_{g,0}p_{g,1}$, $\beta_{g,0}\phi_{g,1}+\beta_{g,1}$, $\phi_{g,T‐1}p_{g,T}$,$p_{g,T}\beta_{g,T‐1}$
$\beta_{g,t},\phi_{g,t},p_{g,t},\Lambda_{g,r,t}$	G	$N_{g}$, $\Lambda_{g,r,t}$, $\phi_{g,1},\cdots ,\phi_{g,T‐2}$,$\beta_{g,2},\cdots ,\beta_{g,T‐2}$, $p_{g,2},\cdots ,p_{g,T‐1}$,
		$\beta_{g,0}p_{g,1}$, $\beta_{g,0}\phi_{g,1}+\beta_{g,1}$, $\phi_{g,T‐1}p_{g,T}$,$p_{g,T}\beta_{g,T‐1}$

**Table A5:** Comparison of intrinsic and extrinsic parameter deficiencies in the southern rock lobster data 1999–2006 and the walleye data 2009–2011

Model	Intrinsic	Extrinsic Deficiency	
	Deficiency	Lobster	Walleye
$\beta_{t},\phi_{g,t},p_{g,t},\Lambda_{g,t}$	0	0	5
$\beta_{t},\phi_{g,t},p_{g,t},\Lambda_{g}$	0	0	5
$\beta_{t},\phi_{g,t},p_{g,t},\Lambda_{t}$	0	0	5
$\beta_{t},\phi_{g,t},p_{g,t},\Lambda$	0	0	5
$\beta_{t},\phi_{g,t},p_{g},\Lambda_{g,t}$	0	0	1
$\beta_{t},\phi_{g,t},p_{g},\Lambda_{g}$	0	0	1
$\beta_{t},\phi_{g,t},p_{g},\Lambda_{t}$	0	0	1
$\beta_{t},\phi_{g,t},p_{g},\Lambda$	0	0	1
$\beta_{t},\phi_{g,t},p_{g},\Lambda_{g,t}$	0	0	2
$\beta_{t},\phi_{g,t},p_{t},\Lambda_{g}$	0	0	2
$\beta_{t},\phi_{g,t},p_{t},\Lambda_{t}$	0	0	2
$\beta_{t},\phi_{g,t},p_{t},\Lambda$	0	0	2
$\beta_{t},\phi_{g,t},p,\Lambda_{g,t}$	0	0	0
$\beta_{t},\phi_{g,t},p,\Lambda_{g}$	0	0	0
$\beta_{t},\phi_{g,t},p,\Lambda_{t}$	0	0	0
$\beta_{t},\phi_{g,t},p,\Lambda$	0	0	0
$\beta_{t},\phi_{g,t},p_{g,t},\Lambda_{g,t}$	0	0	3
$\beta_{t},\phi_{g},p_{g,t},\Lambda_{g}$	0	0	3
$\beta_{t},\phi_{g},p_{g,t},\Lambda_{t}$	0	0	3
$\beta_{t},\phi_{g},p_{g,t},\Lambda$	0	0	3
$\beta_{t},\phi_{g},p_{g},\Lambda_{g,t}$	0	0	0
$\beta_{t},\phi_{g},p_{g},\Lambda_{g}$	0	0	0
$\beta_{t},\phi_{g},p_{g},\Lambda_{t}$	0	0	0
$\beta_{t},\phi_{g},p_{g},\Lambda$	0	0	0
$\beta_{t},\phi_{g},p_{t},\Lambda_{g,t}$	0	0	1
$\beta_{t},\phi_{g},p_{t},\Lambda_{g}$	0	0	1
$\beta_{t},\phi_{g},p_{t},\Lambda_{t}$	0	0	1
$\beta_{t},\phi_{g},p_{t},\Lambda$	0	0	1
$\beta_{t},\phi_{g},p,\Lambda_{g,t}$	0	0	0
$\beta_{t},\phi_{g},p,\Lambda_{g}$	0	0	0
$\beta_{t},\phi_{g},p,\Lambda_{t}$	0	0	0
$\beta_{t},\phi_{g},p,\Lambda$	0	0	0
$\beta_{t},\phi_{g},p_{g,t},\Lambda_{g,t}$	1	1	3
$\beta_{t},\phi_{g},p_{g,t},\Lambda_{g}$	1	1	3
$\beta_{t},\phi_{g},p_{g,t},\Lambda_{t}$	1	1	3
$\beta_{t},\phi_{g},p_{g,t},\Lambda$	1	1	3
$\beta_{t},\phi_{g},p_{g},\Lambda_{g,t}$	0	0	0
$\beta_{t},\phi_{g},p_{g},\Lambda_{g}$	0	0	0
$\beta_{t},\phi_{g},p_{g},\Lambda_{t}$	0	0	0
$\beta_{t},\phi_{g},p_{g},\Lambda$	0	0	0
$\beta_{t},\phi_{t},p_{t},\Lambda_{g,t}$	1	1	2
$\beta_{t},\phi_{t},p_{t},\Lambda_{g}$	1	1	2
$\beta_{t},\phi_{t},p_{t},\Lambda_{t}$	1	1	2
$\beta_{t},\phi_{t},p_{t},\Lambda$	1	1	2
$\beta_{t},\phi_{t},p,\Lambda_{g,t}$	0	0	0
$\beta_{t},\phi_{t},p,\Lambda_{g}$	0	0	0
$\beta_{t},\phi_{t},p,\Lambda_{t}$	0	0	0
$\beta_{t},\phi_{t},p,\Lambda$	0	0	0
$\beta_{t},\phi ,p_{g,t},\Lambda_{g,t}$	0	0	2
$\beta_{t},\phi ,p_{g,t},\Lambda_{g}$	0	0	2
$\beta_{t},\phi ,p_{g,t},\Lambda_{t}$	0	0	2
$\beta_{t},\phi ,p_{g,t},\Lambda$	0	0	2
$\beta_{t},\phi ,p_{g},\Lambda_{g,t}$	0	0	0
$\beta_{t},\phi ,p_{g},\Lambda_{g}$	0	0	0
$\beta_{t},\phi ,p_{g},\Lambda_{t}$	0	0	0
$\beta_{t},\phi ,p_{g},\Lambda$	0	0	0
$\beta_{t},\phi ,p_{t},\Lambda_{g,t}$	0	0	1
$\beta_{t},\phi ,p_{t},\Lambda_{g}$	0	0	1
$\beta_{t},\phi ,p_{t},\Lambda_{t}$	0	0	1
$\beta_{t},\phi ,p_{t},\Lambda$	0	0	1
$\beta_{t},\phi ,p_{t},\Lambda_{g,t}$	0	0	0
$\beta_{t},\phi ,p,\Lambda_{g}$	0	0	0
$\beta_{t},\phi ,p,\Lambda_{t}$	0	0	0
$\beta_{t},\phi ,p,\Lambda$	0	0	0
